# Mucosal adenovirus vaccine boosting elicits IgA and durably prevents XBB.1.16 infection in nonhuman primates

**DOI:** 10.1038/s41590-024-01951-5

**Published:** 2024-09-03

**Authors:** Matthew Gagne, Barbara J. Flynn, Shayne F. Andrew, Josue Marquez, Dillon R. Flebbe, Anna Mychalowych, Evan Lamb, Meredith E. Davis-Gardner, Matthew R. Burnett, Leonid A. Serebryannyy, Bob C. Lin, Zohar E. Ziff, Erin Maule, Robin Carroll, Mursal Naisan, Yogita Jethmalani, Laurent Pessaint, John-Paul M. Todd, Nicole A. Doria-Rose, James Brett Case, Igor P. Dmitriev, Elena A. Kashentseva, Baoling Ying, Alan Dodson, Katelyn Kouneski, Sijy O’Dell, Bushra Wali, Madison Ellis, Sucheta Godbole, Farida Laboune, Amy R. Henry, I-Ting Teng, Danyi Wang, Lingshu Wang, Qiong Zhou, Serge Zouantchangadou, Alex Van Ry, Mark G. Lewis, Hanne Andersen, Peter D. Kwong, David T. Curiel, Mario Roederer, Martha C. Nason, Kathryn E. Foulds, Mehul S. Suthar, Michael S. Diamond, Daniel C. Douek, Robert A. Seder

**Affiliations:** 1 Vaccine Research Center, National Institute of Allergy and Infectious Diseases, National Institutes of Health, Bethesda, MD USA; 2 Department of Pediatrics, Center for Childhood Infections and Vaccines of Children’s Healthcare of Atlanta, Emory University School of Medicine, Atlanta, GA USA; 3 Emory Vaccine Center, Emory University, Atlanta, GA USA; 4 Emory National Primate Research Center, Atlanta, GA USA; 5 Bioqual, Inc., Rockville, MD USA; 6 Department of Medicine, Washington University School of Medicine, St. Louis, MO USA; 7 Department of Radiation Oncology, Washington University School of Medicine, St. Louis, MO USA; 8 Biostatistics Research Branch, Division of Clinical Research, National Institute of Allergy and Infectious Diseases, National Institutes of Health, Bethesda, MD USA; 9 Department of Pathology & Immunology, Washington University School of Medicine, St. Louis, MO USA; 10 Department of Molecular Microbiology, Washington University School of Medicine, St. Louis, MO USA; 11 The Andrew M. and Jane M. Bursky Center for Human Immunology & Immunotherapy Programs, Washington University School of Medicine, St. Louis, MO USA; 12 Center for Vaccines & Immunity to Microbial Pathogens, Washington University School of Medicine, St. Louis, MO USA

**Keywords:** Live attenuated vaccines, Live attenuated vaccines, SARS-CoV-2, Mucosal immunology, Immunological memory

## Abstract

A mucosal route of vaccination could prevent severe acute respiratory syndrome coronavirus 2 (SARS-CoV-2) replication at the site of infection and limit transmission. We compared protection against heterologous XBB.1.16 challenge in nonhuman primates (NHPs) ~5 months following intramuscular boosting with bivalent mRNA encoding WA1 and BA.5 spike proteins or mucosal boosting with a WA1–BA.5 bivalent chimpanzee adenoviral-vectored vaccine delivered by intranasal or aerosol device. NHPs boosted by either mucosal route had minimal virus replication in the nose and lungs, respectively. By contrast, protection by intramuscular mRNA was limited to the lower airways. The mucosally delivered vaccine elicited durable airway IgG and IgA responses and, unlike the intramuscular mRNA vaccine, induced spike-specific B cells in the lungs. IgG, IgA and T cell responses correlated with protection in the lungs, whereas mucosal IgA alone correlated with upper airway protection. This study highlights differential mucosal and serum correlates of protection and how mucosal vaccines can durably prevent infection against SARS-CoV-2.

## Abstract (web summary)

Seder and colleagues show that mucosal adenoviral-vectored vaccine boosting durably prevents infection in nonhuman primates of the highly transmissible, heterologous XBB.1.16 strain of SARS-CoV-2.

## Main

Immunity to severe acute respiratory syndrome coronavirus 2 (SARS-CoV-2) elicited by approved vaccine boosts via the intramuscular (i.m.) route continues to protect against severe disease and hospitalization^1,2^. However, breakthrough infections occur frequently, sustaining the coronavirus disease 2019 (COVID-19) pandemic. Indeed, effectiveness of bivalent mRNA vaccines against infection is limited within 4 months of immunization^3^. Furthermore, reinfections can be associated with postacute sequelae, including long COVID^4^, and the elderly and immunocompromised remain susceptible to severe disease due to poor vaccine responses or diminished innate immunity^5,6^. Thus, deployment of vaccines that prevent or substantially limit breakthrough infections and transmission could have clinical benefit and slow the emergence of new strains.

A vaccine that protects against breakthrough infection needs to overcome four major obstacles: (1) waning of neutralizing antibody titers with time^7–9^, (2) induction of high levels of serum neutralizing antibodies for protection in the nose compared to the lungs^10,11^, (3) emergence of antigenically mismatched variants that exhibit immune escape^12–14^ and (4) antigenic imprinting, which could limit the induction of new variant-specific responses^15–17^.

A vaccine targeted directly to the airways could enhance immunity to SARS-CoV-2 by eliciting or boosting resident memory B cells and plasma cells in the mucosa-associated lymphoid tissue and draining lymph nodes and by locally generating inhibitory IgA^18–20^ and enhancing T cell immunity in the airways. Many intranasal (i.n.) or aerosolized (AE) vaccines are under investigation, including soluble spike (S)^21^ and lipid nanoparticle-encapsulated mRNAs^22^. Virus-vectored vaccines can elicit local humoral and cellular immunity at the site of infection, and current candidates include parainfluenza virus^23^, Newcastle disease virus^24^ and numerous adenovirus serotypes^25–27^. The vaccine ChAd-SARS-CoV-2-S contains the prefusion-stabilized S from the ancestral Wuhan strain inserted into a replication-deficient subgroup E Simian-Ad36 adenovirus and is currently being used in India as a vaccine administered as nasal drops (iNCOVACC)^28^. In animal models, ChAd-SARS-CoV-2-S elicited mucosal and systemic S-specific IgA, IgG and T cell responses and durably protected against multiple SARS-CoV-2 variants in both upper and lower airways^29–33^.

Nonhuman primate (NHP) preclinical models have been important for the development of SARS-CoV-2 vaccines and have been used to demonstrate vaccine-mediated protection against virus variants, define B cell imprinting and provide evidence for the efficacy of boosting^34–39^. However, few NHP studies have assessed how mucosal vaccines using Food and Drug Administration-approved devices for either upper or lower airway delivery influence the durability of immunity and protection^27^ or their effects as a booster following the standard mRNA vaccine regimen used in humans. Although the NHP model for COVID-19 does not fully recapitulate the extent of pathology and inflammation observed in humans with severe disease^40^, immune and virus readouts remain highly predictive of clinical outcomes arising from vaccination and/or challenge with recently circulating Omicron strains^41^. To address the protective role of local humoral and T cell immunity arising from a mucosal boost and determine the optimal delivery route and device, we administered bivalent Wuhan-1 and Omicron BA.5-matched ChAd-SARS-CoV-2-S vaccine to rhesus macaques (*Macaca mulatta*) ~7 months after they had been primed with two i.m. doses of ancestral S-matched mRNA-1273 vaccine. One group of animals received ChAd-SARS-CoV-2-S delivered by a clinically approved aerosol device to the upper and lower airways, whereas another received the vaccine as a mist via the i.n. route using a clinically approved sprayer. For comparison to the available vaccines used in humans at the time of this study, a cohort of animals was boosted via the i.m. route with a matched bivalent (Wuhan-1 and Omicron BA.5 S) mRNA vaccine (mRNA-1273.222). Four and a half months later, all animals were challenged with the highly transmissible heterologous XBB.1.16 strain. We show that mucosal boosting confers durable high-level protection with the potential to block infection and transmission and that there are distinct correlates of protection for mucosal and i.m. vaccines based on tissue-specific immunity in the airways and serum, respectively. We also provide evidence that IgA in the mucosa is both a correlate and potential mechanism of protection in the upper airway.

## Results

### Study design

**Extended Data Fig. 1:**
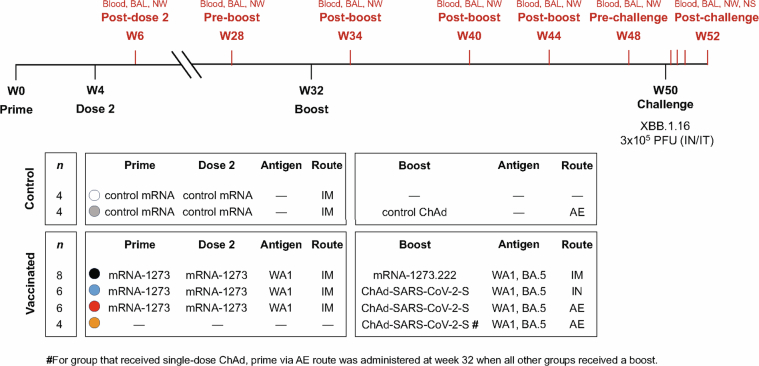
Experimental scheme. NHP (*n* = 8 for control groups and *n* = 24 for vaccine groups) were administered 30 μg of mRNA vaccine via IM route or 1 × 10^11^ virus particles of adenoviral-vectored vaccine via IN or AE route according to immunization schedule shown above. Eighteen weeks after boosting, all primates were challenged with XBB.1.16. Sampling schedule indicated in red.

Twenty Indian-origin rhesus macaques were immunized with 30 µg of Wuhan-1/WA1 S-encoding mRNA-1273 at weeks 0 and 4 by the i.m. route. Dose and regimen were chosen to approximate the immune responses elicited in humans by the standard mRNA-1273 primary series. Seven months later, the 20 NHPs were separated into three groups (Extended Data Fig. 1). One cohort (i.m. boost) of eight NHPs received 30 µg of bivalent mRNA-1273.222, encoding the prefusion-stabilized Wuhan-1 and BA.5 S proteins with two proline-substitution (S-2P), by the i.m. route. This was the benchmark vaccine group for comparing mucosal boosting and was the standard of care in humans for boosting at the time of this study. Another cohort (i.n. boost) of six NHPs received a total of 10^11^ virus particles (vp) composed of an equal mixture of ChAd-SARS-CoV-2-Wuhan-1-S (S-2P) and ChAd-SARS-CoV-2-BA.5-S (S-6P, furin cleavage site mutation)^32^ delivered using the MAD Nasal Intranasal Mucosal Atomization Device (MAD). The MAD is designed to deliver a mist of 30- to 100-µm particles to the upper airway. The final six i.m.-primed NHPs (AE boost) were boosted with the same dose of the bivalent ChAd-SARS-CoV-2-S cocktail as 4-µm particles delivered by AE route via an Investigational eFlow Nebulizer System (PARI Respiratory Equipment). The PARI device is a silicone face mask attachment (PARI SMARTMASK Baby/Kids) to enable particle deposition into the nose and lungs. To investigate the potential of a mucosally delivered virus-vectored vaccine in an unexposed population and allow a comparison between immune responses following boosting and primary immunization, we also administered a single dose of the same bivalent ChAd-SARS-CoV-2-S vaccine to four naive NHPs via aerosol to the nose and lungs (AE prime). As controls, eight naive NHPs (control) received two doses of 30 µg of control mRNA at weeks 0 and 4. At the time of boosting, four of these NHPs received no vaccines, whereas the other four were given 10^11^ vp of a control adenoviral-vectored vaccine (ChAd-Control) by the AE route.

**Extended Data Fig. 2:**
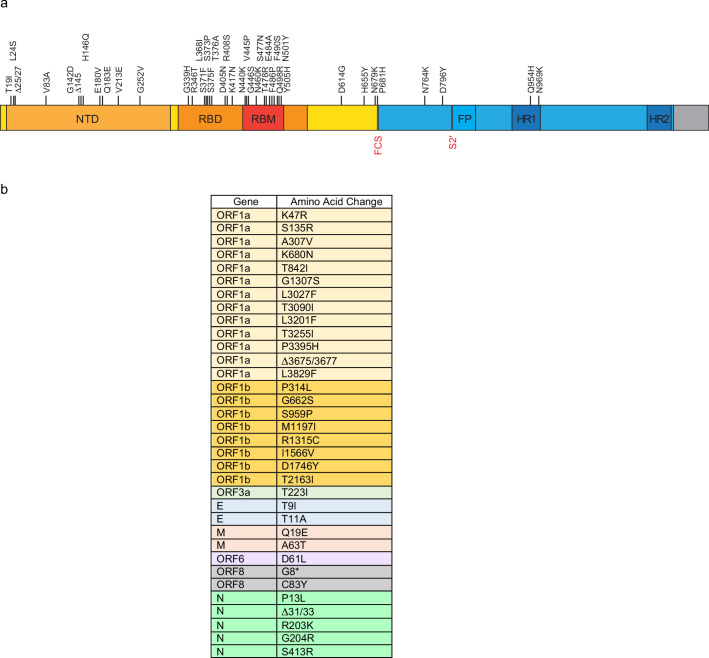
XBB.1.16 sequence. Challenge virus was titered and sequenced to verify presence of canonical XBB.1.16 amino acid substitutions in comparison to ancestral Wuhan-1 strain. Substitutions shown for (**a**) S and (**b**) whole genome. NTD: N-terminal domain. RBD: receptor binding domain. RBM: receptor binding motif. FP: fusion peptide. HR1: heptad repeat 1. HR2: heptad repeat 2. FCS: furin cleavage site. S2′: S2′ site. *: premature stop codon in *Orf8* (common to XBB descendant strains).

Samples from multiple anatomical sites, including bronchoalveolar lavage (BAL) fluid, nasal washes (NWs) and peripheral blood, were collected for analysis at week 6 (postprime peak response), week 28 (preboost), week 34 (postboost peak response), weeks 40 and 44 (memory) and week 48 (prechallenge). At week 50, which was 4.5 months after the boost, all NHPs were challenged with 3 × 10^5^ plaque-forming units (p.f.u.) of XBB.1.16 Omicron strain (Extended Data Fig. 2). Challenge occurred via the i.n. and intratracheal (i.t.) routes. BAL, NWs and nasal swabs (NSs) were collected after the challenge to assess virus replication.

### ChAd-SARS-CoV-2-S limits infection at the site of delivery

**Fig. 1:**
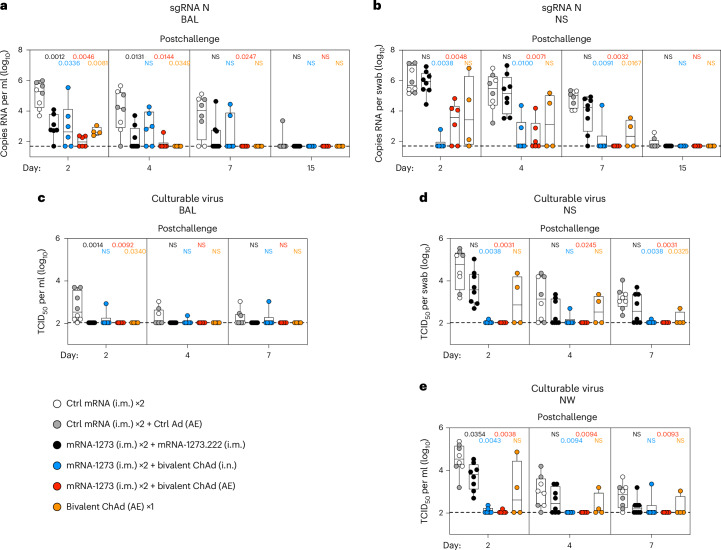
Mucosal adenoviral-vectored vaccine protects against XBB.1.16 replication. NHPs were administered mRNA-1273 or control mRNA at weeks 0 and 4 and were boosted at week 32 with the indicated vaccines. **a**–**e**, Virus replication was measured by detection of sgRNA encoding N (sgRNA N; **a** and **b**), and culturable virus was assessed by TCID_50_ assay (**c–e**) in lower (**a** and **c**) and upper (**b**, **d** and **e**) airways at days 2, 4, 7 and 15 (for sgRNA only). Circles, boxes and horizontal solid lines represent individual animals, interquartile range and median, respectively, whereas minima and maxima are denoted at whisker termini. Assay LOD is represented as a dotted line. Wilcoxon rank-sum tests were conducted for each vaccinated group comparing to pooled controls at the indicated time points. Pairwise two-sided *P* values are shown. NS indicates that comparisons were not significant (*P* > 0.05). Additional details on the statistical analysis are listed in Methods. Immunizations include control mRNA (ctrl mRNA) via the i.m. route, control adenovirus vector (ctrl Ad) via the AE route, mRNA-1273 via the i.m. route, mRNA-1273.222 via the i.m. route and bivalent ChAd-SARS-CoV-2-S via the AE or i.n. route. The numbers of NHPs per group are as follows: control, *n* = 8; i.m. boost, *n* = 8 (except for day 2 in **a**, for which *n* = 7); i.n. boost, *n* = 6; AE boost, *n* = 6; AE prime, *n* = 4.

Subgenomic RNA (sgRNA) encoding nucleoprotein (N) is a highly sensitive measure of virus replication and has been used in prior NHP studies for assessing protection^34,35,42^. In the lower airway (Fig. 1a), sgRNA levels were similar (1.1 × 10^5^ geometric mean copies numbers per ml) between both control cohorts, and the two groups were combined for statistical analysis. On day 2, both the i.m. mRNA and i.n. mucosal boosts yielded significant protection in the lungs, with copy numbers of 9.8 × 10^2^ and 9.5 × 10^2^, respectively, which corresponded to 115-fold reductions compared to the controls (*P* = 0.0012 for i.m. and *P* = 0.0336 for i.n.). The AE boost group showed even greater control of virus replication, with geometric mean titers of 1.0 × 10^2^ (1,100-fold reduction, *P* = 0.0046), which was only marginally higher than the assay limit of detection (LOD). By day 7, nearly 80% (19/24) of the NHPs from any vaccine group (boosted or primed) had cleared sgRNA from the lungs compared to only 25% (2/8) in the control group. However, the difference in sgRNA on day 7 was statistically significant (*P* = 0.0247) only for the AE boost cohort. In summary, all vaccines tested conferred protection in the lower respiratory tract, consistent with clinical data on the efficacy of i.m. vaccines against severe disease^1,43^; however, the AE boost resulted in more rapid and nearly complete control of sgRNA production.

The evolution of virus variants that resist antibody neutralization has resulted in less durable protection against symptomatic upper airway infection, especially from Omicron sublineages^2,44^. Here, geometric mean virus titers in the upper airway (NSs) on day 2 after challenge were 7.3 × 10^5^ in the i.m. mRNA-boosted group and 1.0 × 10^6^ in the controls, a difference of less than twofold (*P* > 0.05); all eight animals in the i.m. boost group had detectable sgRNA in the NSs (Fig. 1b). By contrast, there was a near-complete reduction (14,000-fold, *P* = 0.0038) of sgRNA in the i.n.-boosted group, with geometric mean titers of 7.6 × 10^1^, and levels were measurable in only one of six i.n.-boosted NHPs. sgRNA copy numbers in the NSs of the AE boost group were also significantly lower at 1.8 × 10^3^ (580-fold, *P* = 0.0048). Even in the AE prime group with only four animals there was some evidence of protection, although the effect was not uniform (*P* > 0.05 versus control or i.m. boost). By day 7 after the challenge, essentially all animals in the i.n.- and AE-boosted groups had undetectable sgRNA in the NSs. By contrast, sgRNA copy numbers in the i.m.-boosted group were reduced only tenfold compared to numbers observed in the controls (*P* > 0.05). Thus, even ~5 months after mucosal boosting, heterologous XBB.1.16 virus replication can be effectively controlled in upper and lower respiratory tracts.

We also measured infectious virus by tissue culture infectious dose (TCID)^34^. Like the sgRNA findings, all mucosally vaccinated groups demonstrated similar protection in the lungs as the i.m.-boosted group (*P* > 0.05 versus i.m. boost on all sampling days; Fig. 1c). Although virus was detected in the BAL of seven of eight control animals, only one NHP across all of the vaccinated cohorts had culturable virus. In contrast to the high-level protection in the BAL, significant protection in the nose was observed only for the AE (*P* = 0.0031) and i.n. boost groups (*P* = 0.0038) (Fig. 1d). Of note, there was no control of virus replication in the i.m.-boosted group on day 2 in the nose. Similar findings were obtained in NWs (Fig. 1e).

### Mucosal ChAd-SARS-CoV-2-S elicits durable serum responses

**Fig. 2:**
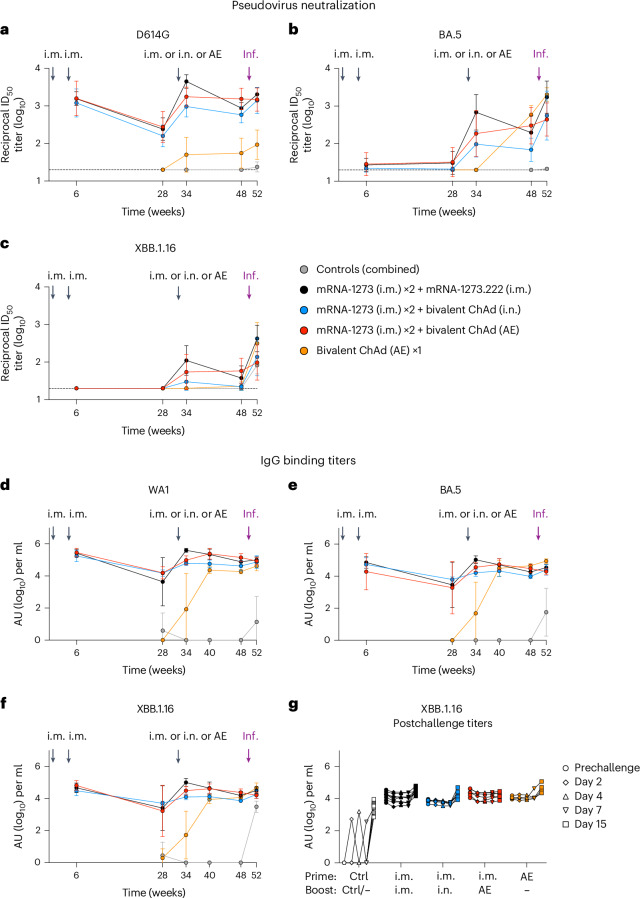
Mucosal adenoviral-vectored vaccine elicits durable systemic humoral responses. NHPs were administered mRNA-1273 or control mRNA at weeks 0 and 4 and were boosted at week 32 with the indicated vaccine. **a**–**g**, Sera were collected postprime (week 6), preboost (week 28), postboost (weeks 34 and 40), prechallenge (week 48) and postchallenge (days 2, 4, 7 and 15). Time of infection (Inf.) is noted by purple arrows. **a**–**c**, Pseudovirus neutralizing responses measured against D614G (**a**), BA.5 (**b**) and XBB.1.16 (**c**). Circles indicate geometric means for each group. Error bars represent geometric standard deviation. The assay LOD is represented as a dotted line. **d**–**f**, Serum IgG binding titers to WA1 (**d**), BA.5 (**e**) and XBB.1.16 S (**f**) at the indicated times. Circles indicate geometric means for each group. Error bars represent geometric standard deviation and may extend beyond the range of the graph. **g**, Postchallenge binding titers to XBB.1.16 S for individual NHPs. Prechallenge samples were collected at week 48. In **g**, lines connect binding titers across time points, while symbols denote AU per ml of individual NHPs. An AU below a value of 1 was replaced with a value of 1 for the data shown in **d**–**g**. Postchallenge fold increases in serum anti-XBB.1.16 IgG binding titers are described in Supplementary Table 1. Immunizations include control mRNA (ctrl mRNA) via the i.m. route, control adenovirus vector (ctrl Ad) via the AE route, mRNA-1273 via the i.m. route, mRNA-1273.222 via the i.m. route and bivalent ChAd-SARS-CoV-2-S via the AE or i.n. route. The number of NHPs per group are as follows: control, *n* = 8; i.m. boost, *n* = 8; i.n. boost, *n* = 6; AE boost, *n* = 6; AE prime, *n* = 4; IC_50_, reciprocal median infectious dose.

**Extended Data Fig. 3:**
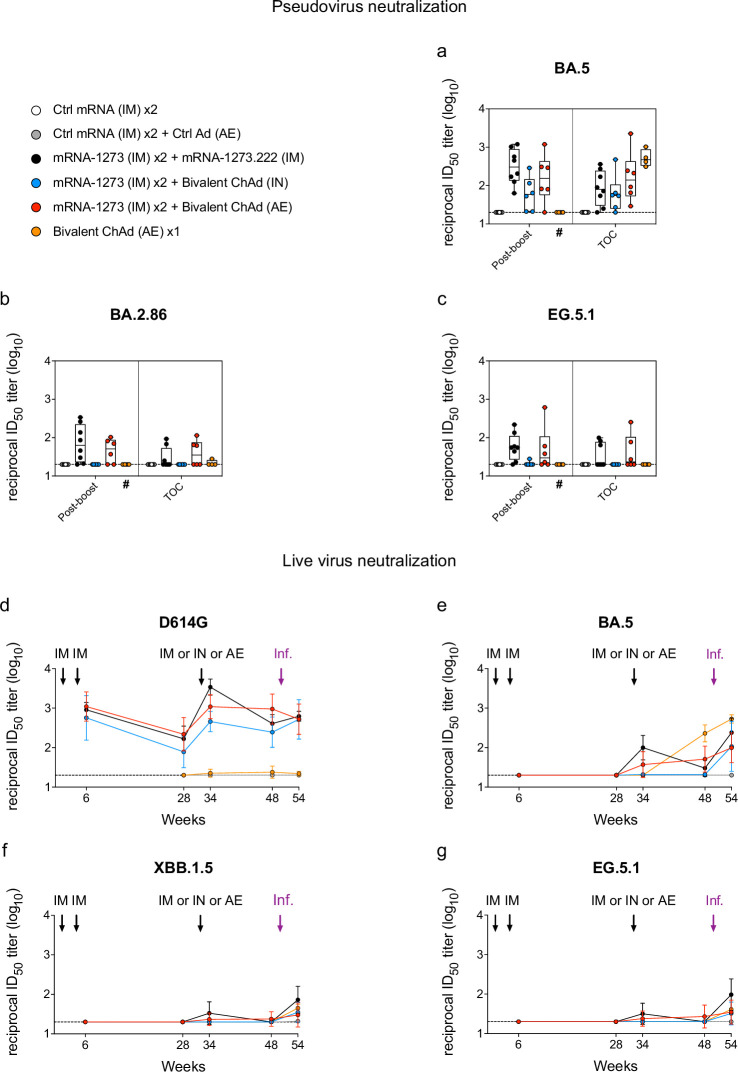
AE or IM boosting elicits durable serum neutralizing responses against emerging SARS-CoV-2 variants. NHP were administered mRNA-1273 or control mRNA at weeks 0 and 4 and boosted at week 32 with the indicated vaccine. (**a-c**) Sera were collected post-boost (week 34) and at the time of challenge (TOC, week 48). Pseudovirus neutralizing responses measured against (**a**) BA.5, (**b**) BA.2.86 and (**c**) EG.5.1. Circles, boxes and horizontal lines represent individual animals, interquartile range and median, respectively, while minima and maxima are denoted at whisker termini. # indicates that while sample collection for AE prime cohort (orange) occurred on week 34, week 34 was two weeks following the single AE prime rather than two weeks following a boost as in other groups. Number of NHP per group (*n*) are as follows: control = 8; IM boost = 8; IN boost = 6; AE boost = 6; AE prime = 4. (**d-g**) Sera were collected post-prime (week 6), pre-boost (week 28), post-boost (week 34), pre-challenge (week 48) and post-challenge (day 28). Neutralizing responses measured against live virus (**d**) D614G, (**e**) BA.5, (**f**) XBB.1.5 and (**g**) EG.5.1. Circles indicate geometric means for each group. Error bars represent geometric standard deviation. Assay LOD in **a-g** represented as dotted line. Number of NHP per group are as follows with *n* listed for all pre-challenge timepoints followed by *n* at day 28 post-challenge in parentheses: control = 8 (4); IM boost = 8 (5); IN boost = 6 (3); AE boost = 6 (3); AE prime = 4 (2).

Antibodies are a primary mechanism of protection against SARS-CoV-2, including neutralizing and Fc effector function activities^10,35,45–48^. Thus, we performed an extensive characterization of humoral responses in blood and respiratory mucosal tissue. Because all boosted groups received the same two-dose mRNA i.m. priming, all preboost data are described as the average across all vaccinated groups, but the group-specific preboost titers are displayed in the figures for reference and used for determination of paired statistical analyses. Virus neutralization was measured using lentiviruses pseudotyped with S from D614G (the benchmark strain), BA.5 or XBB.1.16. Geometric mean serum neutralizing antibody titers to D614G of ~1.4 × 10^3^ reciprocal median infectious dose at week 6 following i.m. priming decreased 6.5-fold to ~2.2 × 10^2^ at week 28 (Fig. 2a), consistent with the waning of antibody titers following an mRNA i.m. prime or boost^35,49^. Following a third i.m. dose (boost), serum titers to D614G were 4.5 × 10^3^ (~20-fold increase), and neutralization breadth also improved with increased titers to BA.5 and XBB.1.16, although responses subsequently waned (Fig. 2a–c). By contrast, although AE or i.n. boosts increased serum neutralization titers to D614G 2 weeks following the boost to 1.8 × 10^3^ and 9.6 × 10^2^, respectively, which were 2.6- and 4.6-fold lower than the i.m.-boosted cohort, titers remained stable over 5 months (Fig. 2a). Indeed, at week 48, titers to D614G in the i.m., i.n. and AE boost groups were 8.7 × 10^2^, 5.8 × 10^2^ and 1.5 × 10^3^, respectively, and to XBB.1.16 were 3.8 × 10^1^, 2.2 × 10^1^ and 5.8 × 10^1^. Neutralizing titers to recently circulating SARS-CoV-2 strains BA.2.86 and EG.5.1 were also assessed (Extended Data Fig. 3a–c). Only the i.m.- and AE-boosted groups had detectable serum neutralizing titers to these variants. Serum antibody titers to authentic live virus showed almost identical patterns as for pseudoviruses, although titers against live viruses were lower (Extended Data Fig. 3d–g).

The AE prime group also exhibited different neutralizing titer kinetics than the i.m. mRNA regimen of two immunizations. Two weeks after the AE prime, neutralizing titers to D614G and BA.5 were 5.0 × 10^1^ and 2.0 × 10^1^, respectively (Fig. 2a,b). Fourteen weeks later, just before challenge, titers to D614G were still at 5.5 × 10^1^, whereas titers to BA.5 had increased 29-fold to 5.9 × 10^2^, indicating that the peak response to the AE vaccine was considerably later than the 2-week interval typically observed following i.m. vaccination. Indeed, at week 48, neutralizing titers to BA.5 were higher in the AE prime group than in all other cohorts, likely reflecting the lack of imprinting to ancestral epitopes used in the mRNA vaccine. Overall, these data are consistent with the findings in human studies that serum neutralizing titers following i.m.-delivered Ad26-vectored vaccines remain stable over time^50,51^.

**Extended Data Fig. 4:**
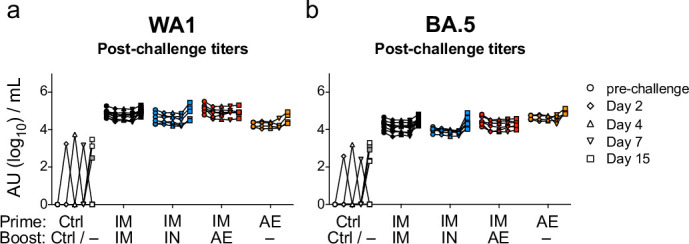
Post-challenge anamnestic responses in sera. NHP were administered mRNA-1273 or control mRNA at weeks 0 and 4 and boosted at week 32 with the indicated vaccine. (**a-b**) Sera were collected pre-challenge (week 48) and post-challenge (days 2, 4, 7 and 15). (**a**) Anti-WA1 S and (**b**) anti-BA.5 S binding titers shown at indicated times. Lines connect binding titers across timepoints while symbols denote AU/mL of individual NHP. AU below a value of 1 were replaced with a value of 1. Number of NHP per group (*n*) are as follows: control = 8; IM boost = 8; IN boost = 6; AE boost = 6; AE prime = 4.

Serum neutralizing titers to XBB.1.16 at 2 weeks postchallenge increased 11.2-fold, 6.2-fold and 13.8-fold in the i.m. boost, i.n. boost and AE prime groups, respectively, whereas titers in the AE boost group increased by only 1.7-fold compared to the time of challenge (Fig. 2c). A kinetic analysis of IgG binding titers showed similar patterns as the neutralizing titers, both pre- and postchallenge (Fig. 2d–f). In control NHPs, primary serum binding IgG responses were detected to all variant S proteins at 2 weeks postchallenge (Fig. 2g and Extended Data Fig. 4), with the highest response against the challenge strain. In comparison, we detected a recall response to XBB.1.16 postchallenge in all vaccinated groups except for the AE boost group, which showed no boost in serum anti-S IgG titers (Fig. 2g). Indeed, titers waned slightly in the AE boost group over the 2-week interval following challenge (Supplementary Table 1), suggesting that rapid control of virus replication in the AE-boosted animals limited antigen availability.

### AE ChAd-SARS-CoV-2-S elicits S-specific BAL B cells

**Fig. 3:**
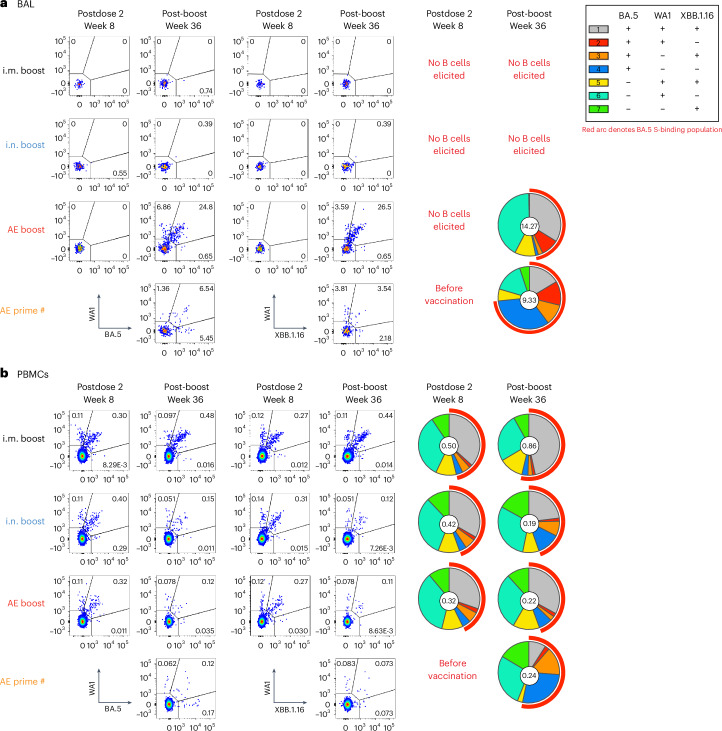
SARS-CoV-2 S-specific memory B cells elicited by vaccination. BAL (**a**) and peripheral blood mononuclear cells (PBMCs; **b**) were collected 4 weeks following the second dose of the primary regimen (week 8) or following a boost (week 36) and were stained with fluorescently labeled WA1, BA.5 and XBB.1.16 S-2P probes. Representative flow cytometry dot plots are shown for memory B cell binding to WA1 and BA.5 probe pairs (first set of columns) and WA1 and XBB.1.16 probe pairs (second set of columns). The pie charts in the last set of columns indicate the proportion of S-binding memory B cells with specificities for various combinations of variants as determined using Boolean gating. The number shown in the center of the pie chart represents the geometric mean frequency of the entire S-binding memory B cell population for all NHPs within the indicated group at the indicated time point. The numbers of NHPs per group are as follows: control, *n* = 8; i.m. boost, *n* = 8; i.n. boost, *n* = 6; AE boost *n* = 6; AE prime, *n* = 4. Pie charts are only provided for groups with S-specific memory B cell frequencies that were clearly distinguished from background staining. Complete longitudinal analysis is shown for all groups in Extended Data Figs. 6 and 7. The number symbol (#) indicates that while sample collection for the AE prime cohort occurred on week 36, week 36 was 4 weeks following the single AE prime rather than 4 weeks following a boost, as in other groups. Complete statistical analyses for comparisons of S-specific memory B cell frequencies are shown in Supplementary Table 2.

**Extended Data Fig. 5:**
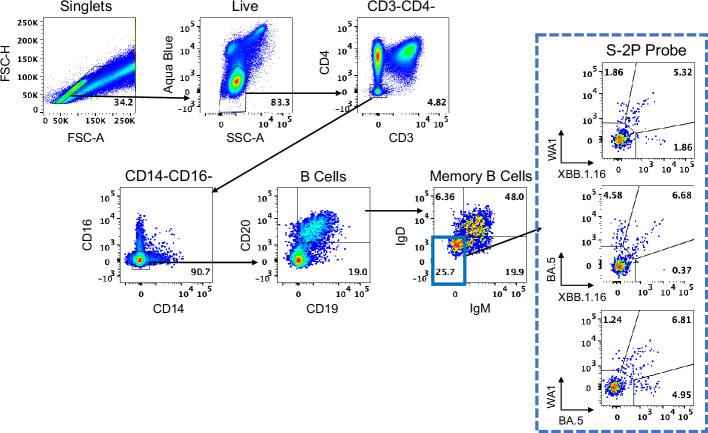
B cell gating strategy. Representative flow cytometry plots showing gating strategy for memory B cells. Cells were gated as singlets and live cells based on forward and side scatter and a live/dead aqua blue stain. B cells were selected based on absence of CD3, CD4, CD14 and CD16 expression and on positive expression of CD20 and CD19. Memory B cells were selected based on lack of surface expression of IgD or IgM. Finally, pairs of variant S-2P probes were used to determine binding specificity.

**Extended Data Fig. 6:**
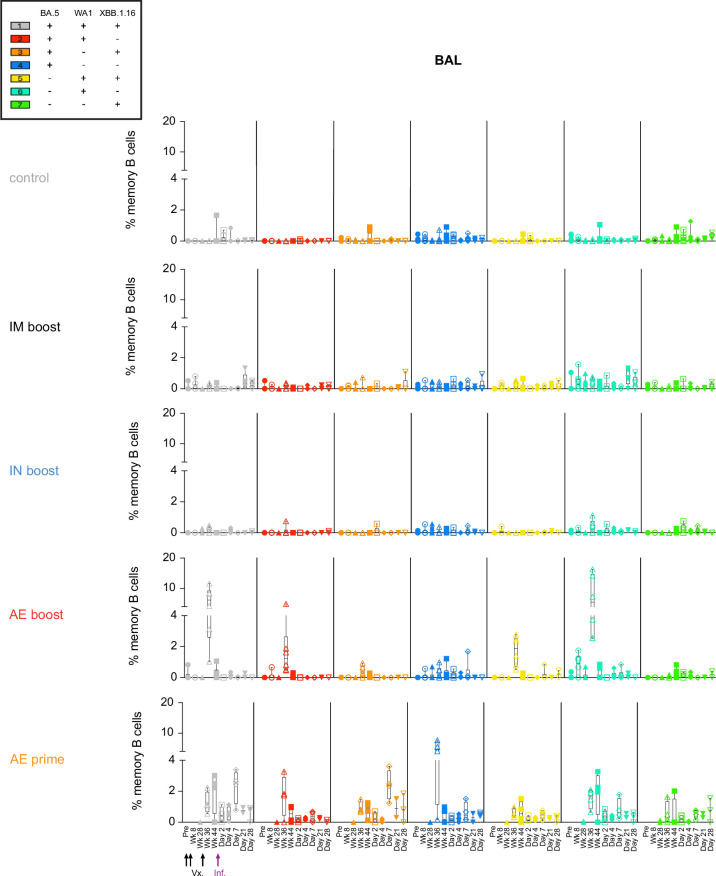
Memory B cell specificity in BAL. BAL was collected prior to vaccination (pre), post-prime (week 8), pre-boost (week 28), post-boost (weeks 36 and 44) and post-challenge (days 2, 4, 7, 21 and 28). Time of vaccinations and challenge indicated by arrows along bottom x-axis. Memory B cells were stained with fluorescently labeled WA1, BA.5 and XBB.1.16 S-2P probes. Symbols represent frequency of variant-specific memory B cells for individual NHP. Colors represent all potential variant-binding combinations as indicated in key at top left of figure. Boxes and horizontal lines represent interquartile range and median, respectively, while minima and maxima are denoted at whisker termini. Break in y-axis indicates a change in scale without a break in the range depicted. Number of NHP per group (*n*) varied based on the timepoint and are listed here, with the corresponding timepoints in parentheses. *n* for control group = 4 (day 21 and 28 post-challenge) and 8 (all preceding timepoints). *n* for IM boost = 7 (day 2 post-challenge), 4 (day 7 post-challenge), 5 (day 21 and 28 post-challenge) and 8 (all other timepoints). *n* for IN boost = 5 (day 2, 4 and 7 post-challenge), 3 (day 21 and 28 post-challenge) and 6 (all other timepoints). *n* for AE boost = 5 (day 2 post-challenge), 3 (day 21 post-challenge), 2 (day 28 post-challenge) and 6 (all other timepoints). *n* for AE prime = 2 (week 28 and day 21 post-challenge), 3 (day 28 post-challenge) and 4 (all other timepoints).

In contrast to circulating serum antibodies, mucosal antibodies would be localized to the sites relevant for initial virus infection and replication. Because airway antibody responses can be mediated by tissue-resident B cells^52,53^, induction or boosting of S-specific memory B cells could be critical to enhancing durable protection. Therefore, we measured the specificity and frequency of mucosal and circulating variant-specific memory B cells using WA1, BA.5 and XBB.1.16 fluorescent S probes (Extended Data Fig. 5). mRNA i.m. vaccination elicited no detectable memory B cells in BAL after the initial primary series (week 8) or following the boost (week 36; Fig. 3a and Extended Data Fig. 6). By contrast, the bivalent ChAd-SARS-CoV-2-S mucosal vaccine elicited S-specific BAL memory B cells at a frequency of 14.3% for the AE boost group and a frequency of 9.3% for the AE prime group at week 36 (4 weeks following immunization) out of all memory B cells (*P* = 0.0007 for the AE boost cohort and *P* = 0.004 for the AE prime cohort compared to the i.m. boost cohort; Supplementary Table 2). The higher frequency observed following the AE boost than that observed following the AE prime, despite the lack of detectable memory B cells before the AE boost, suggests that these B cells might originate from the periphery or from lung-resident B cells that were primed by the i.m. vaccine but were below the LOD in our assays.

**Extended Data Fig. 7:**
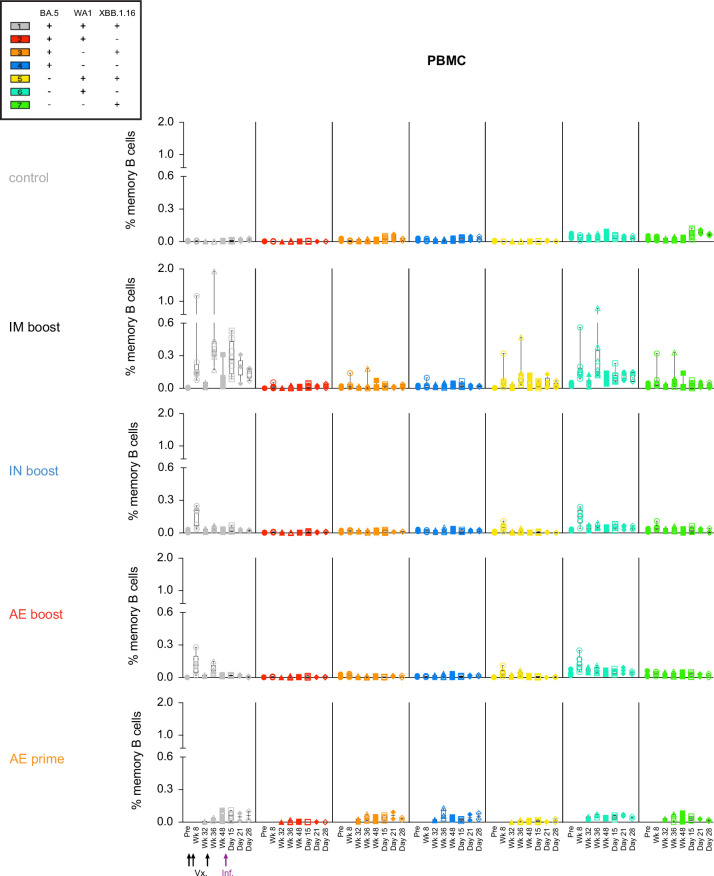
Circulating memory B cell specificity. PBMC were collected prior to vaccination (pre), post-prime (week 8), pre-boost (week 32), post-boost (weeks 36 and 48) and post-challenge (days 15, 21 and 28). Time of vaccinations and challenge indicated by arrows along bottom x-axis. Memory B cells were stained with fluorescently labeled WA1, BA.5 and XBB.1.16 S-2P probes. Symbols represent frequency of variant-specific memory B cells for individual NHP. Colors represent all potential variant-binding combinations as indicated in key at top left of figure. Boxes and horizontal lines represent interquartile range and median, respectively, while minima and maxima are denoted at whisker termini. Break in y-axis indicates a change in scale without a break in the range depicted. Number of NHP per group are as follows with *n* first listed for pre-challenge and early post-challenge timepoints followed by *n* for days 21 and 28 post-challenge in parentheses: control = 8 (4); IM boost = 8 (5); IN boost = 6 (3); AE boost = 6 (3); AE prime = 4 (2).

In the blood, mRNA i.m. priming elicited S-binding memory B cells with a frequency of ~0.4% at week 8 (Fig. 3b). This declined to ~0.15% by week 32 (Extended Data Fig. 7). mRNA i.m. boosting increased the frequency to 0.86% at week 36, which was higher than after the primary immunization series (*P* = 0.0078; Supplementary Table 2). Of note, neither AE nor i.n. boosting increased the frequency of circulating memory B cells in the blood compared to the postprime frequency.

Previous studies by us and others have shown that initial priming with mRNA imprints the B cell response such that B cells specific for the priming antigen are recalled after subsequent heterologous antigenic exposure^16,17,36,54,55^. We found that B cell specificity imprinted by the initial mRNA vaccination remained unchanged following any of the boosts. B cells were predominately cross-reactive to multiple variants or were specific for WA1, which was the priming immunogen. Indeed, the proportion of memory B cells that recognized epitopes unique to BA.5 did not increase following the bivalent boost, despite inclusion of BA.5 S. Furthermore, there were insufficient numbers of memory B cells in the BAL of the i.m. and i.n. boost cohorts to enable characterization of binding specificity (Fig. 3a), which reflected the limited capacity of those vaccines to elicit lower airway immune responses. By contrast, AE priming elicited a population of memory B cells in both the blood and the lungs that were specific for BA.5 S and represented 26.2% of the entire peripheral S-binding population and an even larger proportion of the mucosal B cells (Fig. 3). Following challenge with XBB.1.16, cross-reactive B cell responses were recalled in the i.m. boost and AE prime groups, whereas XBB.1.16-specific responses were elicited in the control animals. Strikingly, there was no expansion of the S-specific memory B cell population in the mucosally boosted groups (Extended Data Figs. 6 and 7).

### Analysis of upper and lower airway antibody responses

**Fig. 4:**
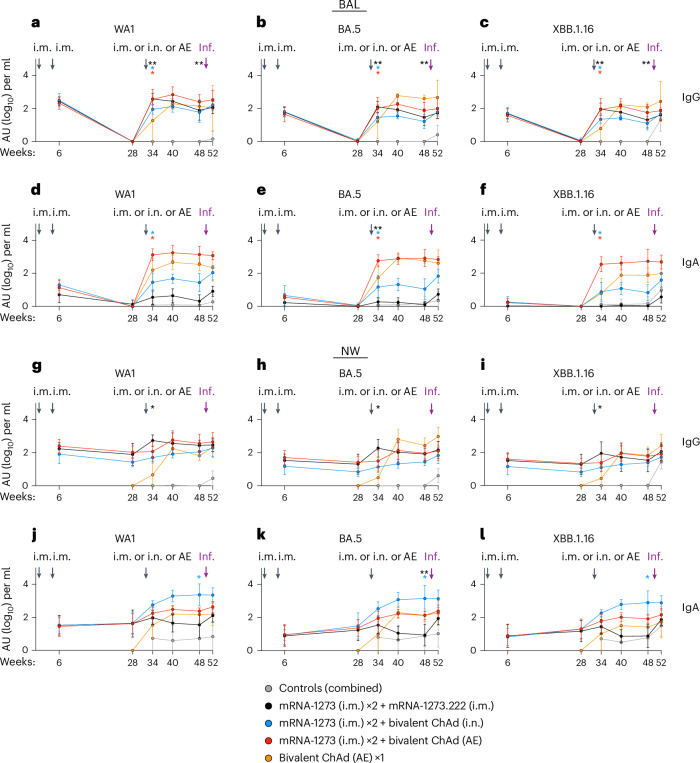
Mucosal IgG and IgA responses following vaccination. **a**–**l**, NHPs were administered mRNA-1273 or control mRNA at weeks 0 and 4 and were boosted at week 32 with the indicated vaccine. BAL (**a**–**f**) and NWs (**g**–**l**) were collected postprime (week 6), preboost (week 28), postboost (weeks 34 and 40), prechallenge (week 48) and 2 weeks postchallenge (week 52). Time of infection (Inf.) is noted by purple arrows. IgG (**a**–**c** and **g**–**i**) and IgA (**d**–**f** and **j**–**l**) binding titers were measured to WA1, BA.5 and XBB.1.16 S as indicated. Circles indicate geometric means for each group. Error bars represent geometric standard deviation and may extend beyond the range of the graph. AU values below 1 were replaced with a value of 1. Wilcoxon signed-rank tests were conducted for anti-S titers at week 34 (postboost) versus week 28 (preboost) and also for week 48 (prechallenge) versus week 34 (postboost) for each vaccinated group. Additional details on statistical analyses and corresponding fold changes in geometric mean serum neutralizing antibody titer are available in Supplementary Table 3. Asterisks (*) indicate pairwise two-sided *P* values (*P < 0.05 and **P < 0.01). All other comparisons were not significant (*P* > 0.05). Immunizations included control mRNA (ctrl mRNA) via the i.m. route, control adenovirus vector (ctrl Ad) via the AE route, mRNA-1273 via the i.m. route, mRNA-1273.222 via the i.m. route and bivalent ChAd-SARS-CoV-2-S via the AE or i.n. route. The numbers of NHPs per group are as follows: control, *n* = 8; i.m. boost, *n* = 8; i.n. boost, *n* = 6; AE boost, *n* = 6; AE prime, *n* = 4.

mRNA vaccines administered by the i.m. route principally elicit S-specific serum and mucosal IgG with minimal production of mucosal IgA^56,57^. Indeed, anti-S IgG responses were boosted in the BAL following a third i.m. dose of mRNA, with titers to WA1 increasing ~380-fold (*P* = 0.0078) from ~1 × 10^0^ at week 28 to 3.8 × 10^2^ arbitrary binding units (AU) per ml at week 34 (2 weeks postboost; Fig. 4a). BAL IgG titers to BA.5 and XBB.1.16 also increased significantly (*P* = 0.0078 for both variants; Fig. 4b,c and Supplementary Table 3a). However, IgA titers in the lungs to WA1 S and all other variants after i.m. boost were low (Fig. 4d–f). By contrast, the AE boost strikingly increased both IgG (~360-fold increase, *P* = 0.0313) and IgA titers (~1,200-fold increase, *P* = 0.0313; Supplementary Table 3b) to WA1 S in the BAL, and these titers were stable over the following 5 months (*P* > 0.05). This stability contrasts with the rapid waning observed in mucosal IgG binding titers elicited by the i.m. boost (*P* = 0.0078 for all variants; Supplementary Table 3a). In the i.n. boost group, there were modest increases in anti-WA1 S titers in BAL, with fold increases of ~90-fold for IgG and ~26-fold for IgA, compared to the preboost time point with no subsequent waning (*P* = 0.0313 and *P* > 0.05 for expansion and contraction time points, respectively, for all variants). Last, 2 weeks after the AE prime (week 34), we also detected WA1 S-specific IgG (1.9 × 10^1^) and IgA (1.6 × 10^2^) in the BAL, and these continued to rise until week 40, similar to the trend observed for serum neutralizing antibody titers. However, the changes observed in the AE prime cohort were not significant (*P* > 0.05), possibly due to the small size of the cohort.

In the NWs, each of the vaccine boosts resulted in modest increases in anti-S IgG binding titers (Fig. 4g–i), in contrast to the large increases observed in the BAL with a significant effect observed only for the i.m. boost cohort (*P* = 0.0391 for WA1 and XBB.1.16 and *P* = 0.0156 for BA.5; Supplementary Table 3c). The AE prime group, however, induced upper airway WA1 S-specific IgG that reached titers equivalent to the mucosally boosted groups by week 40. Importantly, the i.n. boost increased anti-S IgA levels in the nose by week 34 with ~13-fold, 15-fold and 10-fold increases to WA1, BA.5 and XBB.1.16, respectively, which were higher than in all other groups. IgA titers did not decline between weeks 34 and 48 and instead significantly increased approximately fourfold for all variants (*P* = 0.0313). IgA titers also increased following an AE boost or prime, although these changes were not significant (Fig. 4j–l and Supplementary Table 3d). Anti-S IgA titers remained stable in the mucosal groups until challenge.

### Kinetics of airway mucosal antibody responses postchallenge

**Extended Data Fig. 8:**
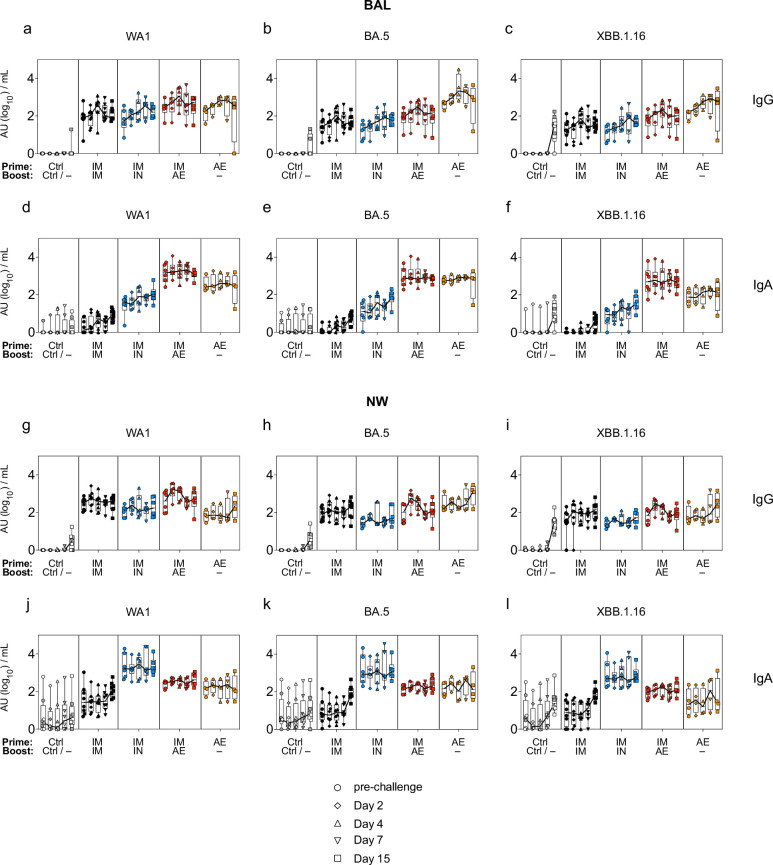
XBB.1.16 challenge elicits rapid anamnestic antibody responses in the BAL of vaccinated NHP. NHP were administered mRNA-1273 or control mRNA at weeks 0 and 4 and boosted at week 32 with the indicated vaccine. (**a-f**) BAL and (**g-l**) NW were collected pre-challenge (week 48) and on days 2, 4, 7 and 15 post-challenge. (**a-c, g-i**) IgG and (**d-f, j-l**) IgA binding titers measured to WA1, BA.5 or XBB.1.16 S as indicated. Symbols indicate individual NHP. Boxes and thin horizontal lines represent interquartile range and median, respectively, while minima and maxima are denoted at whisker termini. Thick solid lines connect median binding titers across timepoints. AU below a value of 1 were replaced with a value of 1. Number of NHP per group (*n*) are as follows: control = 8; IM boost = 8; IN boost = 6; AE boost = 6; AE prime = 4.

Antibody protection in the lung can be mediated through neutralizing or Fc effector function activities^10,45–48^. Moreover, in prior NHP studies, we also showed that there was a rapid anamnestic antibody response in BAL within 2–4 days of SARS-CoV-2 challenge of i.m.-vaccinated animals^35,58^, coincident with control of virus replication. Here, there was an increase in anti-S IgG in BAL postchallenge across all the vaccine groups (Extended Data Fig. 8a–c). By day 4 postchallenge, geometric mean IgG binding titers in the BAL to XBB.1.16 increased three- to fivefold for all vaccinated groups compared to the prechallenge time points (Extended Data Fig. 8c), although this response was transient in the AE boost group. Furthermore, anamnestic IgA responses in BAL to any variant S were only clearly detected in the i.m.- and i.n.-boosted cohorts, with delayed kinetics compared to IgG (Extended Data Fig. 8d–f). Anti-XBB.1.16 S IgA binding titers increased 3.4-fold in the i.m. boost group and 5.8-fold in the i.n. boost group by day 15 postchallenge compared to the prechallenge time point (Extended Data Fig. 8f). Despite the anamnestic responses in the lower airway of the i.n.-boosted group, there were no corresponding recall responses in the nose. Indeed, NW IgA was boosted by XBB.1.16 challenge only in the i.m.-boosted group by day 15 (tenfold; Extended Data Fig. 8l). This is consistent with persistent virus replication in the nose observed only in the i.m.-boosted group.

### Differential antibody neutralization in the nose and lungs

**Fig. 5:**
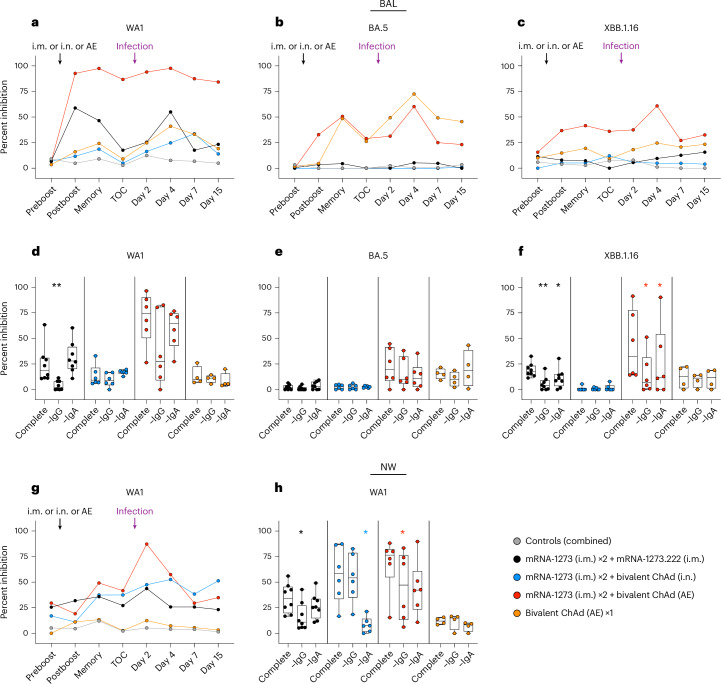
Functional IgG and IgA responses in the upper and lower airways following vaccination. **a**–**h**, NHPs were administered mRNA-1273 or control mRNA at weeks 0 and 4 and were boosted at week 32 with the indicated vaccine. BAL (**a**–**c**) and NWs (**g**) were collected preboost (week 28), postboost (week 34), at a memory time point (week 40) and at time of challenge (TOC; week 48) and postchallenge (days 2, 4, 7 and 15). WA1 (**a** and **g**), BA.5 (**b**) and XBB.1.16 (**c**) S binding to ACE2 was measured with and without the addition of mucosal fluids to determine percent inhibition as a surrogate for neutralizing antibodies. Symbols in **a**–**c** and **g** indicate median percent inhibition of each group. Mucosal fluids at a late memory time point prechallenge (week 44) from BAL (**d**–**f**) or NWs (**h**) were used to inhibit WA1 (**d** and **h**), BA.5 (**e**) or XBB.1.16 (**f**) S binding to ACE2 as complete fluid or after the selective depletion of either IgG or IgA. Symbols in **d**–**f** and **h** represent individual NHPs. Boxes and horizontal lines represent interquartile range and median, respectively, while minima and maxima are denoted at whisker termini. Wilcoxon signed-rank tests were conducted for ACE2–S binding inhibition at week 44 with complete or depleted mucosal fluids. Asterisks (*) indicate pairwise two-sided *P* values (*P < 0.05 and **P < 0.01). All other comparisons were not significant (*P* > 0.05). Additional details on statistical analyses and corresponding fold changes in median percent inhibition are provided in Supplementary Table 4. Immunizations include control mRNA (ctrl mRNA) via the i.m. route, control adenovirus vector (ctrl Ad) via the AE route, mRNA-1273 via the i.m. route, mRNA-1273.222 via the i.m. route and bivalent ChAd-SARS-CoV-2-S via the AE or i.n. route. The numbers of NHPs per group are as follows: control, *n* = 8; i.m. boost, *n* = 8; i.n. boost, *n* = 6; AE boost, *n* = 6; AE prime, *n* = 4.

To determine the potential contributions of IgG and IgA to protection in the respiratory tract, we measured the capacity of BAL or NW antibodies to inhibit binding between variant S and its angiotensin-converting enzyme 2 (ACE2) receptor as a functional surrogate of neutralization^59^. ACE2 binding inhibition activity in lung and nasal fluids at week 28 (preboost) was low to undetectable for all groups following two i.m. doses of mRNA-1273 (Fig. 5). In the i.m. boost group, median inhibition of WA1 binding in the BAL increased 2 weeks after the boost from ~6.9% to 58.8% (*P* = 0.0078; Fig. 5a and Supplementary Table 4a). Binding inhibition then waned (*P* = 0.0078 for week 48 versus week 34), consistent with the decrease in serum neutralization and binding titers. We did not observe a clear increase in inhibition of any other SARS-CoV-2 variant after i.m. boosting (Fig. 5a–c).

The AE boost also increased ACE2 binding inhibition in BAL, with median inhibition against WA1 rising to 92.7% 2 weeks after the boost (*P* = 0.0313) but with no evidence of waning (*P* > 0.05). Inhibition of XBB.1.16 binding also increased (~8% to 36.7%) but did not reach significance (Fig. 5c). The AE prime group elicited ACE2 binding inhibition, although this was principally directed to BA.5, possibly reflecting the more immunogenic 6P-stabilized structure of the BA.5 S than the 2P-stabilized Wuhan S (Fig. 5b). In comparison, the i.n. boost only modestly increased ACE2 binding inhibition in BAL fluid against XBB.1.16 with little or no effect on inhibition of other variants.

In the NWs, preboost median ACE2 binding inhibition to WA1 was ~21.3% and only marginally different 2 weeks after any boost (Fig. 5g and Supplementary Table 4b). However, by the memory time point at week 40, binding inhibition had increased to 35.8%, 37.4% and 49.2% in the i.m., i.n. and AE boost groups, respectively, reflecting different antibody induction kinetics in the upper and lower airways. Virus challenge elicited a recall response in all boosted groups by days 2–4.

### Role of IgG and IgA for functional inhibition in airways

To assess the potential mechanisms of mucosal antibody responses, we assessed the relative contributions of IgG and IgA to neutralization in mucosal fluids by measuring ACE2 binding inhibition after in vitro depletion of either IgG or IgA. Samples were collected at week 44, a postboost memory time point. In BAL, IgA depletion had limited effects on inhibitory responses in any group except for a modest reduction for anti-XBB.1.16 following an i.m. or AE boost, although the reduction was more apparent for the latter group (Fig. 5d–f and Supplementary Table 4c). However, IgG depletion reduced median WA1 S–ACE2 binding inhibition in BAL from 18.9% to 2.2% in the i.m.-boosted group (*P* = 0.0078). Although IgG depletion did not result in a significant reduction in inhibition of WA1 binding for the AE-boosted group, likely due to the wide range of responses across the cohort, we observed a significant decrease in functional antibodies to XBB.1.16 S (*P* = 0.0313; Fig. 5d). All other groups showed limited binding inhibition.

In the upper airway, IgG depletion substantially reduced WA1 S–ACE2 binding inhibition in the i.m.-boosted group from 34.3% to 10.9%, whereas IgA depletion had a lesser effect, reducing inhibition to 25.5% (*P* = 0.0156 and *P* > 0.05 for IgG and IgA, respectively; Fig. 5h and Supplementary Table 4d). For the AE-boosted group, both IgG and IgA contributed to ACE2 binding inhibition, with inhibition declining from 76.5% to 47.1% and 42.2%, respectively, although the effect was only significant for IgG depletion (*P* = 0.0313). Importantly, the i.n.-boosted group was unique in that IgA depletion almost eliminated binding inhibition, suggesting that functional activity of antibodies in the nose of this group was predominately IgA-mediated (*P* = 0.0313).

### Mucosal ChAd-SARS-CoV-2-S vaccine expands S-specific T cells

**Fig. 6:**
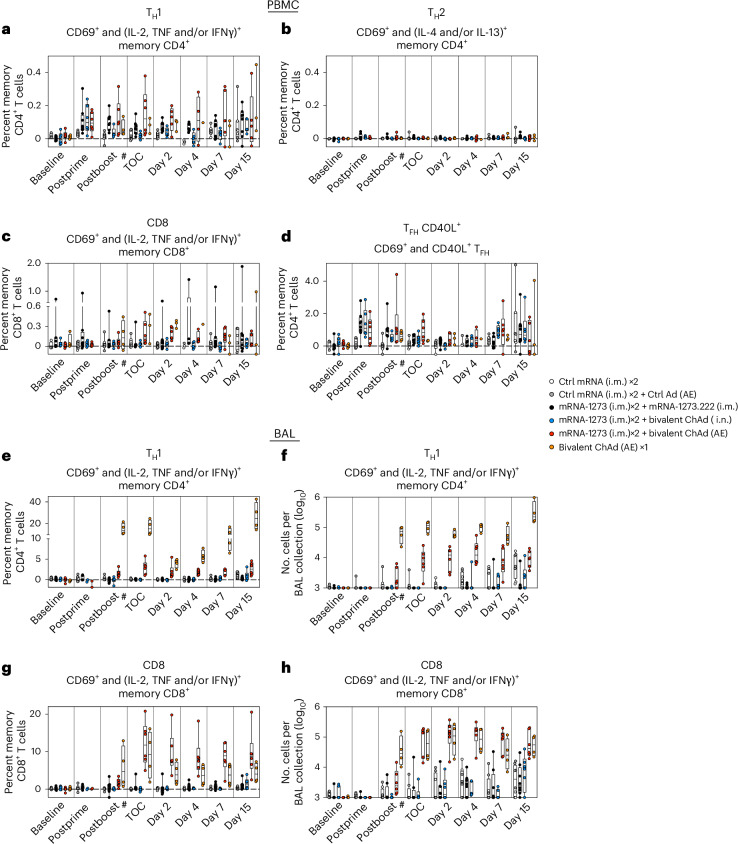
AE immunization elicits durable CD4^+^ and CD8^+^ T cell responses in BAL. **a**–**h**, PBMCs (**a**–**d**) and BAL fluid (**e**–**h**) were collected before vaccination (baseline) and at weeks 6 (postprime), 34 (postboost) and 48 (time of challenge) as well as on days 2, 4, 7 and 15 postchallenge. Lymphocytes were stimulated with SARS-CoV-2 S1 and S2 peptide pools (WA1) and were then measured by intracellular staining. **a**,**b**,**e**, Percentage of memory CD4^+^ T cells with T_H_1 markers (IL-2, TNF or IFNγ; **a** and **e**) or type 2 helper T (T_H_2) cell markers (IL-4 or IL-13; **b**) following stimulation. **c**,**g**, Percentage of memory CD8^+^ T cells expressing IL-2, TNF or IFNγ following stimulation. **d**, Percentage of T_FH_ cells that express CD40L following stimulation. Breaks in the *y* axis indicate a change in scale without a break in the range depicted. Dotted lines are set at 0%. Fold changes in S-specific T cell frequencies are provided in Supplementary Table 5. **f**,**h**, Absolute numbers of S-reactive T_H_1 CD4^+^ (**f**) or CD8^+^ T cells (**h**) in the BAL. Counts below a value of 1 (due to background subtraction) were replaced with a value of 1 for data in **f** and **h**. Circles, boxes and horizontal lines in **a**–**h** represent individual animals, interquartile range and median, respectively, while minima and maxima are denoted at whisker termini. Reported values may be negative due to background subtraction and may extend below the range of the *y* axis. The number symbol (#) indicates that while sample collection for the AE prime cohort (orange) occurred on week 34, week 34 was 2 weeks following the single AE prime rather than 2 weeks following a boost, as in all other groups. Immunizations include control mRNA (ctrl mRNA) via the i.m. route, control adenovirus vector (ctrl Ad) via the AE route, mRNA-1273 via the i.m. route, mRNA-1273.222 via the i.m. route and bivalent ChAd-SARS-CoV-2-S via the AE or i.n. route. The numbers of NHPs per group are as follows: control, *n* = 8; i.m. boost, *n* = 8; i.n. boost, *n* = 6; AE boost, *n* = 6; AE prime, *n* = 4. Due to prespecified minimum cell numbers per sample required for analysis, some time points include data from fewer NHPs than the full group size.

**Extended Data Fig. 9:**
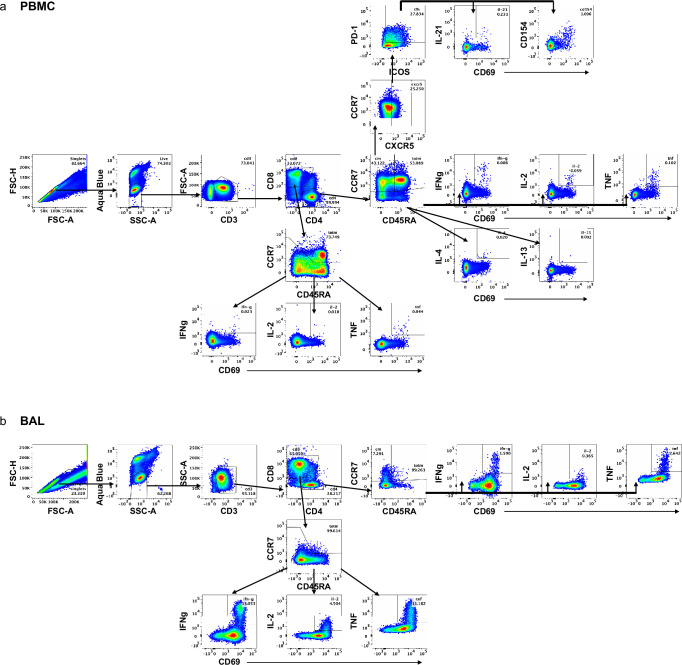
T cell gating strategy. Representative flow cytometry plots showing gating strategy for ICS with (**a**) PBMC or (**b**) BAL lymphocytes following S or N peptide stimulation. Cells were gated as singlets and live cells on forward and side scatter and a live/dead aqua blue stain. CD3^+^ events were gated as CD4^+^ or CD8^+^ T cells. Total memory CD8^+^ T cells were selected based on expression of CCR7 and CD45RA. SARS-CoV-2-specific memory CD8^+^ T cells were gated according to co-expression of CD69 and IL-2, TNF or IFNγ. The CD4^+^ events were defined as naïve, total memory or central memory according to expression of CCR7 and CD45RA. CD4^+^ cells with a T_H_1 phenotype were defined as memory cells that co-expressed CD69 and IL-2, TNF or IFNγ. For PBMC only, CD4^+^ cells with a T_H_2 phenotype were defined as memory cells that co-expressed CD69 and IL-4 or IL-13, whereas T_FH_ cells were defined as central memory CD4^+^ T cells that expressed CXCR5, ICOS and PD-1. T_FH_ cells were further characterized as IL-21^+^, CD69^+^ or CD40L^+^, CD69^+^.

mRNA or adenoviral vaccines administered via the i.m. route induce CD4^+^ and CD8^+^ T cell responses against SARS-CoV-2, which may contribute to protection^60–63^. Thus, we assessed the kinetics of T cell responses in blood and BAL (Extended Data Fig. 9). Following a boost with the i.m. mRNA vaccine or mucosal adenoviral-vectored vaccine, there were no further increases in the frequency of SARS-CoV-2-specific type 1 helper T (T_H_1), follicular helper T (T_FH_) or CD8^+^ T cell subsets in the blood above those elicited by the two-dose primary series (Fig. 6a–d). However, in the BAL, there was an increase in S-specific T_H_1 and CD8^+^ T cells (coexpressing CD69 and interferon-γ (IFNγ), tumor necrosis factor (TNF) or interleukin-2 (IL-2)) following the AE boost (Fig. 6e,g and Supplementary Table 5). The T cell response was highest in the AE prime group, which had the greatest frequency of S-specific T_H_1 and CD8^+^ T cells, with median values of 14.1% and 4.7%, respectively, at week 34 compared to 1.2% and 1.4% in the AE boost group, although the frequency of S-specific CD8^+^ T cells in the AE boost group did increase to 11.8% by week 48. For the i.m., i.n. and control groups, the median frequencies of S-specific T_H_1 and CD8^+^ T cells in the BAL were all less than 1% at both weeks 34 and 48. This suggests that antigen presentation in the lungs and mucosa-associated lymphoid tissue is an especially efficient way to induce T cell responses in BAL.

We further interrogated the impact of vaccination route on nonspecific T cell recruitment into the lungs (Supplementary Fig. 1). Interestingly, i.m. priming with mRNA, but not boosting, had a suppressive effect on total CD4^+^ and CD8^+^ T cells, perhaps reflective of immune cell efflux from the BAL and recruitment to the initial site of antigen presentation. Although we observed a trend toward greater CD4^+^ and CD8^+^ T cell counts following AE priming with the ChAd-SARS-CoV-2-S vaccine as compared to the other vaccine regimens, this effect was not noticeably different than what was observed in the controls, including both controls that were primed with mRNA only and those that were given control ChAd-vector via the AE route. The relative increase in total T cells following an AE prime compared to the i.m. or i.n. boost (<1 log) could also not account for the dramatic increase (~2 log) in antigen-specific T cells (Fig. 6f,h).

**Extended Data Fig. 10:**
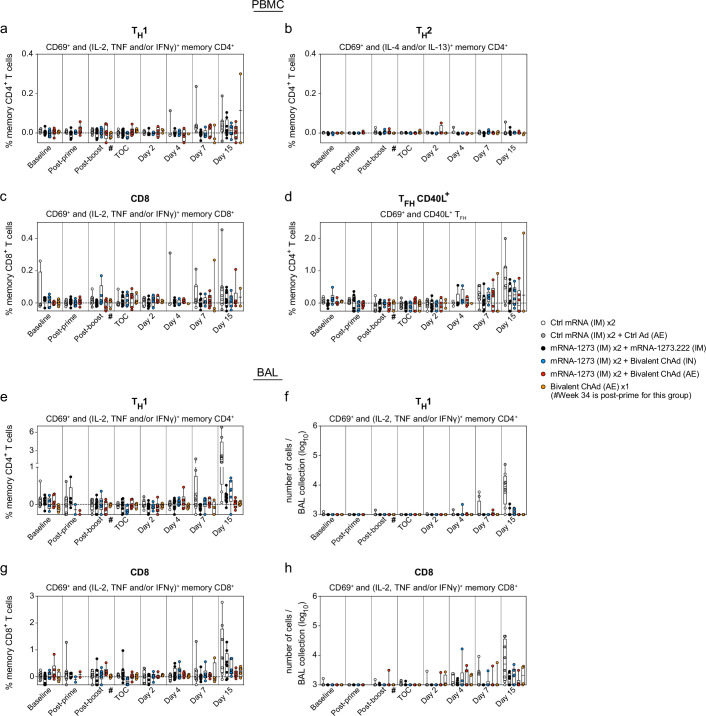
T cell responses to N peptides in PBMC and BAL following IM or mucosal boosting. (**a-d**) PBMC and (**e-h**) BAL fluid were collected pre-vaccination (baseline) and at weeks 6 (post-prime), 34 (post-boost) and 48 (time of challenge, TOC) as well as on days 2, 4, 7 and 15 post-challenge. Lymphocytes were stimulated with SARS-CoV-2 N peptides (WA1) and then measured by intracellular staining. Percentage of memory CD4^+^ T cells with (**a, e**) T_H_1 markers (IL-2, TNF or IFNγ) or (**b**) T_H_2 markers (IL-4 or IL-13) following stimulation. (**c, g**) Percentage of memory CD8^+^ T cells expressing IL-2, TNF or IFNγ following stimulation. (**d**) Percentage of T_FH_ cells that express CD40L following stimulation. Break in y-axis in **e** indicates a change in scale without a break in the range depicted. Dotted lines set at 0%. Absolute number of N-reactive (**f**) T_H_1 CD4^+^ or (**h**) CD8^+^ T cells in the BAL are also depicted. Counts below a value of 1 (due to background subtraction) were replaced with a value of 1 for data in **f** and **h**. Circles, boxes and horizontal lines in **a-h** represent individual animals, interquartile range and median, respectively, while minima and maxima are denoted at whisker termini. Reported values may be negative due to background subtraction and may extend below the range of the y-axis. # indicates that while sample collection for AE prime cohort (orange) occurred on week 34, week 34 was two weeks following the single AE prime rather than two weeks following a boost as in all other groups. Number of NHP per group (*n*) are as follows: control = 8; IM boost = 8; IN boost = 6; AE boost = 6; AE prime = 4. Due to pre-specified minimum cell numbers per sample required for analysis, some timepoints include data from fewer NHP than the full group size.

As T cells may contribute to vaccine-mediated protection against severe SARS-CoV-2 disease in the lung^32,64,65^, we next assessed the magnitude of the antigen-specific T cell compartment in the BAL following virus challenge. There was no significant increase in the number of S-specific T cells in the AE boost group at any time or in the AE prime group until day 15, after virus had been cleared from the lungs; there was a trend toward a small increase in antigen-specific lung T cell counts in the i.m. and i.n. boost groups beginning at day 4, consistent with an i.t.-delivered infection that bypasses the localized protective effect of an i.n.-administered vaccine (Fig. 6f,h). Responses to the N protein, which was not incorporated into the vaccine, were highest in the control group, followed by the i.m. boost group (Extended Data Fig. 10), whereas cohorts that received mucosal vaccines had low to undetectable N-specific responses, consistent with rapid suppression of virus replication.

### Immune correlates of virus replication after challenge

Based on the extensive B and T cell assessment, we analyzed multiple parameters to establish criteria associated with reduction of virus replication in the airways. Given the limited number of animals in each vaccine-specific group, we analyzed all animals together. In previous studies, immune correlates have been typically defined only at the peak of response shortly after vaccination when serum antibody titers are highest^10,11,66^. We found that multiple humoral immune measures at both the peak time after boosting (week 34) and immediately before challenge (week 48) were inversely correlated with virus replication in the lower airways as measured by sgRNA copy number in the BAL (Table 1). These included neutralizing and binding antibody titers in the sera, mucosal IgG and IgA titers in the lungs, ACE2-binding inhibitory antibodies in the BAL and frequency of lung S-specific T_H_1 and CD8^+^ T cells. In addition, we constructed multiple parametric linear models to predict virus titers as a function of both vaccination group and one of the various immune readouts (with each measure incorporated into its own individual model) among all animals. The linear model incorporating anti-XBB.1.16 IgG titers in the blood at week 48 was sufficiently predictive of virus copy number in the lungs such that the vaccination group provided no additional predictive power. Importantly, and in contrast to the correlations in the BAL, only NW IgA titers were associated with reduction in virus replication in the upper airway (*P* < 0.001 for titers at week 48).

**Table 1:** Immune correlates of virus replication after challenge

Variable	Week 34 (postboost)		Week 48 (TOC)	
	*ρ*	*P*	*ρ*	*P*
Lower airway correlations	Versus BAL sgRNA N on day 2 postchallenge			
Serum pseudovirus neutralization (XBB.1.16)	−0.429	**0.016**	−0.502	**0.004**
Serum IgG (anti-XBB.1.16)^a^	−0.525	**0.002**	−0.652	**<0.001**
BAL IgG (anti-XBB.1.16)	−0.503	**0.004**	−0.625	**<0.001**
BAL IgA (anti-XBB.1.16)	−0.493	**0.005**	−0.590	**<0.001**
BAL ACE2 binding inhibition (XBB.1.16)	−0.499	**0.004**	−0.301	0.100
BAL S-specific T_H_1 cells	−0.226	0.221	−0.422	**0.018**
BAL S-specific CD8^+^ T cells	−0.446	**0.012**	−0.574	**0.001**
Upper airway correlations	Versus NS sgRNA N on day 2 postchallenge			
Serum pseudovirus neutralization (XBB.1.16)	0.087	0.638	−0.182	0.318
Serum IgG (anti-XBB.1.16)	−0.045	0.808	−0.202	0.268
NW IgG (anti-XBB.1.16)	−0.149	0.415	−0.297	0.099
NW IgA (anti-XBB.1.16)	−0.354	**0.047**	−0.591	**<0.001**
NW ACE2 binding inhibition (WA1)	0.050	0.784	−0.228	0.210

Immune parameters measured following the boost (week 34) and immediately before challenge (week 48) were assessed for potential correlations with virus sgRNA N copy numbers at day 2 postchallenge in BAL and NSs. Spearman’s rank correlation coefficients (*ρ*) and two-sided *P* values are listed for each association, with significant associations shown in bold. No adjustments were made for multiple comparisons. A linear model was also constructed to predict virus sgRNA copy number as a function of vaccination group and various immune readouts one by one to determine which immune parameters were independently predictive of virus load.

^a^Week 48 values predictive of virus titers in the linear model such that vaccine status is no longer informative.

## Discussion

In this study, we evaluated whether targeting a SARS-CoV-2 vaccine directly to the mucosa could boost upper airway immunity such that durable protection could be achieved against a heterologous highly transmissible immune-evading variant. To achieve optimal mucosal boosting, we compared two different delivery devices that are both approved for use in humans for either vaccines or other indications. Our principal findings were (1) tissue-specific induction of airway mucosal antibodies is dependent on vaccine delivery route; (2) antigen-specific immunoglobulin class is dependent on the site of antigen encounter, with i.m. vaccines primarily boosting systemic IgG and mucosal vaccines inducing both mucosal IgG and IgA; (3) immunity induced by mucosal adenoviral-vectored vaccines was durable over a period of 5 months, in contrast to the rapid peak and subsequent waning typical of i.m. vaccination with mRNA vaccines; and (4) control of virus infection in the lower airway, upper airway and both compartments was characteristic of i.m., i.n. and AE vaccination, respectively. Additionally, our data suggest that IgA is a correlate and potential mechanism of protection in the upper airway that is distinct and not reflected by serum neutralizing titers.

These data indicate that vaccines directed at both the lungs and nose (AE groups) can induce broad multicompartment mucosal immunity. This response effectively and rapidly suppresses virus replication such that insufficient antigen is available to promote systemic recall responses. However, mucosal vaccination directed primarily at the nose (i.n. boost group), although capable of boosting upper airway IgA titers and preventing local virus replication, did not elicit antigen-specific memory B cells in BAL nor suppress virus replication in the lungs as effectively as the AE boost. This highlights that the form of mucosal delivery can optimize immune responses in specific upper and lower airway compartments.

The prevention or substantive inhibition of most respiratory infections is mediated by antibodies in the airways. For instance, mucosal IgG antibodies specific to SARS-CoV-2 S are readily elicited by i.m. mRNA vaccination and correlate with protection^10,34,67^. By contrast, i.m. mRNA vaccines against SARS-CoV-2 elicit low to undetectable secretory IgA responses^20,35,57^, although IgA is associated with protection from upper airway infection for SARS-CoV-2, influenza virus and respiratory syncytial virus^19,20,68–70^. In this study, i.n. delivery of adenoviral vaccines primarily induced upper airway IgA rather than IgG, which may be due to activation of IgA-producing plasmablasts and/or memory B cells in the nasal-associated lymphoid tissue^71,72^. Thus, enhanced IgA production by targeted vaccine delivery could overcome the limited protection against infection with current SARS-CoV-2 variants provided by i.m.-elicited neutralizing IgG^10,11,73^. Indeed, it is notable that serum neutralizing antibody titers to recently circulating variants EG.5.1 and BA.2.86 were detectable following i.m., but not i.n., boosting and that these responses had no association with protection in the upper airway, suggesting that secretory IgA, and not serum neutralizing IgG, is the likely mechanism and correlate of protection following i.n. immunization. It is also notable that although we measured total antigen-specific IgA, additional analysis indicates that this is comprised of dimeric secretory IgA. Collectively, these data suggest that there may be distinct correlates of protection for mucosally and i.m.-delivered vaccines. Mucosal vaccines may require assessment of antibody or T cell responses in the upper airways to correlate with protection rather than the standard neutralizing antibody correlate in the blood from currently available i.m.-delivered vaccines.

Virus-vectored vaccines also effectively induce T cell responses^23,32,60^. We showed that AE-delivered ChAd-vectored vaccines induced high frequencies of CD4^+^ and CD8^+^ T cells in BAL that were sustained, which is consistent with prior studies in NHPs using AE delivery of other antigens^74–76^. Regarding the role of T cells in mediating protection in this study, it is notable that the frequency of S-specific T cells after challenge did not markedly increase until after virus replication had been controlled in animals that were primed or boosted by AE adenoviral vaccine or i.m. mRNA. Reductions in virus replication in BAL at day 2 postchallenge in animals boosted by i.n. vaccination was evident even though there were low to undetectable T cell responses. Although we observed a correlation between S-specific T cell frequency and virus titers in the lungs, the rapid and complete control of virus replication in the upper and lower airways by day 2 are consistent with an antibody-mediated mechanism of protection. Nevertheless, the induction of T cell responses in the lungs following AE boosting or priming could provide another layer of immunity in the context of further antigenic shift centered on B cell epitopes^62,77,78^. In addition, the high numbers of total T cells, including antigen-specific T_H_1 cells, which were elicited by the AE prime in particular, may suggest a unique benefit for an AE rather than i.m. prime in the case of a future novel respiratory pathogen.

A key aspect of optimizing mucosal immune responses in humans by vaccination will be the delivery device. We chose two approaches selectively targeting the upper (i.n.) and lower airways (AE) using devices with extensive safety data in humans. The eFlow Technology used in the Investigational eFlow Nebulizer System (PARI) for AE delivery has been approved for treatment of cystic fibrosis^79^ and, as shown here, is highly efficient for generating robust antibody and T cell responses in the lung^74–76^. The MAD device for i.n. delivery is similar to an i.n. sprayer used to administer the live attenuated quadrivalent influenza vaccine^80^. Furthermore, the ChAdOx1 nCov-19 (AZD1222) vaccine, which uses a different adenovirus serotype than ChAd-SARS-CoV-2-S, was administered using the MAD device in a phase 1 clinical trial for safety and immunogenicity^25^. In contrast to our data, the ChAdOx1 nCov-19 clinical study showed limited immunogenicity. This may be due to one or more factors, including (1) the use of a different simian adenovirus strain with possibly distinct tropism, (2) a higher vaccine dose (1 × 10^11^ vp of ChAd-SARS-CoV-2-S compared to 5 × 10^9^–5 × 10^10^ for ChAdOx1) and (3) the prefusion stabilization of S for ChAd-SARS-CoV-2-S compared to wild-type S protein (used in ChAdOx1), as stabilization is more efficient at inducing neutralizing responses against class I fusion proteins^81–83^. Similarly, a recent report comparing i.n. and i.t. (via endoscopy) delivery of Ad26.COV-2.S in NHPs after priming with i.m.-delivered adenovirus showed significant to complete protection in the lower airway by i.t. administration but more limited protection in the upper airway. Moreover, there was no protective effect for i.n. administration in the upper or lower airway^27^. Of note, we evaluated virus replication by measurement of sgRNA encoding the N protein, a substantially more sensitive assessment of virus replication than the transcript encoding the E protein, which was used in this other study, and we substantiated our findings with virus growth assessment. Differences in the adenovirus serotypes or dose, S inserts, vaccine used for priming and amount and strain of challenge virus may explain some of the differences observed in protection between these studies.

The selection of immunization route and mucosal delivery device for limiting infection in humans may depend on how SARS-CoV-2 is transmitted. Although virus is likely emitted as small aerosol droplets (<5 μm) generated deep in the lower airways^84^, it remains unclear which anatomical site in the upper respiratory tract is the primary site of infection after natural exposure. Although we did not achieve full protection in the lungs following i.n. boosting via MAD, it is notable that we experimentally administered our challenge stock via both i.n. and i.t. routes. If natural infection occurs primarily in the nose, then delivery of challenge virus directly into the trachea may have hindered the ability of the i.n. vaccine to completely block infection.

### Limitations of the study

First, although we model the impact of prior immune exposure to multiple antigens (WA1 and BA.5), we did not attempt to recapitulate the antecedent exposures that humans now have including infections and additional vaccine doses. However, two mRNA vaccinations likely induce some level of immune imprinting^17,36^. Second, although we included the AE prime group to characterize the role of prior immunity, we did not include a similar i.n. prime arm because of limited numbers of NHPs. Likewise, we did not have a control group for i.n. adenovirus exposure. As the control AE group had no effect on protection, it is probable that an i.n. control would have likewise exerted no effect on virus replication. Finally, we did not administer ChAd-SARS-CoV-2-S via the i.m. route, as the major comparison was with currently available i.m. mRNA vaccines; however, a direct comparison of adenoviral-vectored vaccines delivered via the i.m. and AE routes could allow us to distinguish features of immunogenicity associated with route of administration.

This study provides a proof of principle for mucosal vaccination in a relevant preclinical model to achieve durable cross-variant immunity with essentially complete prevention of XBB.1.16 infection. It is possible that alternative platforms could achieve similar results if delivered to the mucosa. Although there are rodent data for i.n. delivery of protein and mRNA^21,22^, there are more limited NHP data modeling prior immunity by widely used mRNA vaccines before mucosal boosting to demonstrate durable protection against infection. Indeed, protein and mRNA may need to be formulated specifically to enhance penetration into the upper airway mucosal tissue^85^, which is a natural feature of many vaccines derived from viruses that have evolved for this purpose. Furthermore, the choice of adenovirus serotype may be relevant, as there may be distinct preferences for replication in the upper airway compared to the gut^86^. It is likely that our findings regarding the role of mucosal IgA as a correlate of protection in the upper airway may also pertain to alternative mucosal delivery platforms and that measurement of these responses rather than serum neutralizing titers would be key for clinical development and authorization. The data presented here provide a roadmap for next-generation vaccines against SARS-CoV-2 and other respiratory pathogens with pandemic potential to achieve near-sterilizing prevention of infection and transmission blockade and potentially limit the rate of virus evolution. This approach also has the potential to reduce global virus burden and alter the outbreak dynamics of disease.

## Methods

### Rhesus macaque model

All experiments were conducted according to National Institutes of Health (NIH) standards on the humane care and use of laboratory animals, and all procedures were approved by, and were conducted in accordance with, regulations of the Animal Care and Use Committees of the NIH Vaccine Research Center (VRC) and Bioqual. Animals were housed under animal biosafety level 2 conditions for the immunization phase. Up to 1 week before (for acclimation) and during the challenge phase, animals were housed under animal biosafety level 3 conditions, per Bioqual facility standard operating procedures.

At the time of study enrollment, 2- to 6-year-old male Indian-origin rhesus macaques were primed with mRNA vaccine or were administered control mRNA. Animals were later stratified into groups for vaccine boosting based on age and weight.

### Preclinical mRNA and adenoviral-vectored vaccines

A sequence-optimized mRNA encoding prefusion-stabilized SARS-CoV-2 S protein containing two proline stabilization mutations (S-2P)^83,87^ for Wuhan-1 or bivalent Wuhan-1/BA.5 was synthesized in vitro and formulated^88^. The ChAd-SARS-CoV-2-BA.5-S vector expressed a prefusion-stabilized S glycoprotein of BA.5 (GenBank QJQ84760; T19I, L24S, 25–27del, 69–70del, G142D, V213G, G339D, S371F, S373P, S375F, T376A, D405N, R408S, K417N, N440K, G446S, L452R, S477N, T478K, E484A, F486V, Q498R, N501Y, Y505H, D614G, H655Y, N679K, P681H, N764K, D796Y, Q954H and N969K) containing six proline substitutions (F817P, A892P, A899P, A942P, K986P and V987P) and furin cleavage site substitutions (RRARS to GSASS, residues 682–686) as described elsewhere^89^. Control mRNA ‘NTFIX-01 (not translated factor 9)’ was synthesized and similarly formulated into lipid nanoparticles as previously described^37^.

The ChAd-SARS-CoV-2-S replication-incompetent vector (Simian-Ad36) encoding the prefusion-stabilized SARS-CoV-2 S-2P and empty ChAd-Control vector were generated as described previously^29^. The ChAd-SARS-CoV-2-BA.5-S genome was rescued following transfection of the T-REx-293 cell line (Invitrogen, R710-07). Replication-incompetent ChAd-SARS-CoV-2-BA.5-S, ChAd-SARS-CoV-2-S and ChAd-Control vectors were scaled up in HEK-293 cells (ATCC, CRL-1573) and purified by CsCl density-gradient ultracentrifugation. Viral particle concentrations were determined by spectrophotometry at 260 nm, as previously described^90^.

### Vaccine delivery to rhesus macaques

For i.m. delivery, mRNA was administered in 1 ml of formulated lipid nanoparticles diluted in PBS into the right quadricep, as previously described^34,35,91^. For AE delivery, each animal was administered 1 ml of adenoviral-vectored vaccine (diluted in PBS to a concentration of 1 × 10^11^ vp) via a pediatric silicone face mask (PARI SMARTMASK Baby/Kids) attached to an Investigational eFlow Nebulizer System (PARI) that delivered 4-μm particles deep into the lungs, as previously described^92^. For i.n. delivery, each animal was administered 200 µl of adenoviral-vectored vaccine as 30- to 100-μm particles into each nostril for a total volume of 400 µl (diluted in PBS to a concentration of 2.5 × 10^11^ vp) via MAD Nasal (Teleflex).

### Immunization and challenge schedule

Twenty NHPs were primed at weeks 0 and 4 with two doses of 30 µg of mRNA-1273 via the i.m. route. At week 32, the following groups received a third dose: (1) eight NHPs (i.m. boost) received 30 µg of mRNA-1273.222 via the i.m. route, (2) six NHPs (i.n. boost) received 10^11^ vp of the bivalent cocktail of ChAd-SARS-CoV-2-Wuhan-1-S and ChAd-SARS-CoV-2-BA.5-S^32^ via the i.n. route, and (3) six NHPs (AE boost) received 10^11^ vp of the bivalent cocktail of ChAd-SARS-CoV-2-S via the AE route. Also, at week 32, a naive cohort of four NHPs (AE prime) were administered 10^11^ vp of the bivalent cocktail of ChAd-SARS-CoV-2-S via the AE route.

Finally, an additional group of eight NHPs received two doses of 30 µg of control mRNA via the i.m. route at weeks 0 and 4. At week 32, four of these NHPs received 10^11^ vp of control ChAd via the AE route.

All NHPs were challenged at week 50 (18 weeks after final immunization) with 3 × 10^5^ p.f.u. of SARS-CoV-2 Omicron XBB.1.16. The virus inoculum was administered as 2.25 × 10^5^ p.f.u. in 3 ml via the i.t. route and 0.75 × 10^5^ p.f.u. in 1 ml via the i.n. route (MAD) with a volume of 0.5 ml distributed evenly into each nostril.

### Isolation and sequencing of the XBB.1.16 challenge stock

XBB.1.16 (EPI_ISL_17417328) was isolated from a residual NS kindly provided by B. Pinsky (Stanford University). Virus was plaque purified and propagated once in VeroE6-TMPRSS2 cells to generate a working stock. XBB.1.16 stock was then sequenced as previously described^34,36^. Briefly, NEBNext Ultra II RNA Prep reagents and multiplex oligonucleotides (New England Biolabs) were used to prepare Illumina-ready libraries, which were sequenced on a MiSeq (Illumina). Demultiplexed sequence reads were analyzed in the CLC Genomics Workbench v.23.0.1 by (1) trimming for quality, length and adaptor sequence, (2) mapping to the Wuhan-Hu-1 SARS-CoV-2 reference (GenBank NC_045512), (3) improving the mapping by local realignment in areas containing insertions and deletions and (4) generating both a sample consensus sequence and a list of variants. Default settings were used for all tools.

### Cell lines

Cell lines used for ChAd production included the T-REx-293 cell line (Invitrogen, R710-07) for rescue of the ChAd-SARS-CoV-2-BA.5-S genome and HEK-293 cells (ATCC, CRL-1573) for scaling up of ChAd vectors.

VeroE6-TMPRSS2 cells were used for propagation of XBB.1.16 challenge stock and live virus neutralization assays, while Vero-ACE2/TMPRSS2 cells were used for TCID_50_ assays. Both cell lines were provided by A. Creanga (VRC/National Institute of Allergy and Infectious Disease (NIAID)) and were cultured in complete DMEM as previously described^93^. The 293T-human ACE2 cell line (obtained from M. Farzan and H. Mu at the University of Florida Scripps Institute) was used for pseudovirus neutralization assays.

### sgRNA quantification

sgRNA was isolated and quantified by researchers blinded to vaccine status, as previously described^34^. Briefly, total RNA was extracted from BAL fluid and NSs using an RNAzol BD column kit (Molecular Research Center). PCR reactions were conducted with TaqMan Fast Virus 1-Step Master Mix (Applied Biosystems), forward primer in the 5′ leader region and N gene-specific probe and reverse primer as previously described^36^. The following primers and probes were used: sgLeadSARSCoV2_F (5′-CGATCTCTTGTAGATCTGTTCTC-3′), N2_P (5′-FAM-CGATCAAAACAACGTCGGCCCC-BHQ1-3′) and wtN_R (5′-GGTGAACCAAGACGCAGTAT-3′). Amplifications were performed with a QuantStudio 6 Pro Real-Time PCR System (Applied Biosystems). The assay lower LOD was 50 copies per reaction.

### TCID_50_ quantification

Infectivity of virus was determined by TCID_50_ assay on Vero-ACE2/TMPRSS2 cells. One hour before the assay, the growth medium was removed from the cells and replaced with 180 μl per well of 2% cDMEM (DMEM supplemented with 2% fetal bovine serum (FBS), 2 mM l-glutamine and 1× antibiotic). Samples were serially diluted tenfold in DMEM containing 2% FBS. Twenty microliters of diluted sample was added to cells in quadruplicate. After 5 days, infectious medium was removed, and cells were fixed and stained with crystal violet (20% methanol and 2.5 g of crystal violet). Virus replication was scored as a lack of crystal violet staining. TCID_50_ values were calculated using the Reed–Muench method^94^. The lower LOD for the quantification of virus titer was 108.

### Pseudovirus neutralization

SARS-CoV-2 lentiviral pseudotyped neutralization assays were performed on integrated automation platforms consisting of a Biomek liquid handler from Beckman Coulter, an ambient temperature labware hotel (Thermo Scientific), a 37 °C incubator (Thermo Scientific) and a Molecular Devices Paradigm Multimode reader, as previously described^36^. The automated assay methods were operated using Beckman Coulter SAMI EX software (version 5.0). On day 1, samples were diluted starting at 1:20 and then serially diluted fourfold (seven times) in D10 culture medium (10% FBS, DMEM and 0.3 µl ml^–1^ puromycin) in a bulk sterile polypropylene 384-well deep-well plate. The diluted samples were transferred from bulk dilution plates into individual 384-well black tissue culture plates at 30 µl per well (Thermo Scientific Nunc 384-well polystyrene plates, cell culture surface, 164564). SARS-CoV-2 S-pseudotyped viruses were diluted in D10 and added at 30 µl per well into tissue culture plates containing the serially diluted samples, followed by a 45-min incubation at 37 °C and 5% CO_2_. 293T-human ACE2 reporter cells were added at a concentration of 10,000 cells per well in 20 µl into virus/sample tissue culture plates, followed by a 72-h incubation at 37 °C and 5% CO_2_. On day 4 (72 h from day 1), 50 µl of culture medium was removed from the plates, and 30 µl of luciferase substrate (PerkinElmer Britelite Plus, 6066769) was added. The plates were incubated at room temperature for 2 min, and the samples/luciferase were mixed. The luminescence signal (relative luminescence unit (RLU)) was measured using a Paradigm Multimode reader. The neutralization percentage of the test sample was determined by normalization of the test sample RLU to the RLU of virus and cell control wells with the following calculation: percentage = [(test wells – average of cell control wells) – (average of virus control wells – average cell control wells)] ÷ (average virus control wells – average cell control wells) × 100. The neutralization curve fit was generated on a NAB analysis module on the Labkey web-based server with five-parameter nonlinear regression. Neutralizing antibody titers are expressed as the reciprocal of the serum dilution required to reduce RLU by 50% and are reported as inhibition dosage.

### Live virus neutralization

D614G^95^ and BA.5 (ref. ^96^) variants have been previously described. XBB.1.5 was provided by A. Pekosz (Johns Hopkins University), whereas EG.5.1 (EPI_ISL_17977757) was isolated from a residual NS kindly provided by B. Pinsky. All variants were plaque purified and propagated once in VeroE6-TMPRSS2 cells to generate working stocks. Viruses were deep sequenced and confirmed as previously described^97^.

Focus reduction neutralization assays were performed as previously described^98^. Briefly, samples were diluted starting at 1:10 in a total volume of 60 μl and serially diluted threefold (eight times) using DMEM. Serially diluted samples were incubated with an equal volume of SARS-CoV-2 (100–200 foci per well) at 37 °C for 1 h in a round-bottom 96-well culture plate. The antibody–virus mixture was then added to Vero-TMPRSS2 cells and incubated at 37 °C for 1 h. After incubation, the antibody–virus mixture was removed, and 100 µl of prewarmed 0.85% methylcellulose overlay was added to each well. Plates were then incubated at 37 °C for 18 to 40 h. The methylcellulose overlay was removed, and cells were washed six times with PBS. Cells were fixed with 2% paraformaldehyde in PBS for 30 min. Following fixation, plates were washed twice with PBS, and permeabilization buffer (0.1% bovine serum albumin and 0.1% Saponin in PBS) was added to permeabilize cells for at least 20 min. Cells were incubated with primary anti-SARS-CoV-2 S directly conjugated to Alexa Fluor-647 (clone CR3022-AF647, Cell Signaling, 37475; 1:5,000) overnight at 4 °C. Cells were then washed twice with PBS and imaged on an ELISpot reader (CTL Analyzer).

Antibody neutralization was quantified by counting the number of foci for each sample using the Viridot program^99^. The neutralization titers were calculated as 1 – (ratio of the mean number of foci in the presence of sera and foci at the highest dilution of the respective sera sample). Each specimen was tested in duplicate. The half-maximal focus reduction neutralization titers were interpolated using a four-parameter nonlinear regression in GraphPad Prism 9.2.0. Samples that did not neutralize at the LOD at 50% were plotted at 20 and were used for geometric mean and fold change calculations.

### Serum and mucosal antibody binding

Quantification of antibodies was performed using multiplex electrochemiluminescence serology assays by Meso Scale Discovery (MSD), as previously described^36,91^. Briefly, total antigen-specific antibodies were measured by MSD V-Plex SARS-CoV-2 Panel 36var3 for S (kindly provided by MSD), according to the manufacturer’s instructions, except that 25 μl of sample and detection antibody were used per well. Heat-inactivated plasma was diluted 1:10,000 in Diluent 100. BAL and NW fluid were concentrated tenfold with Amicon Ultra centrifugal filter devices with a 30-kDa molecular weight cutoff (MWCO; Millipore Sigma). Concentrated BAL samples were subsequently diluted 1:100 for IgG and 1:50 or 1:100 for IgA using Diluent 100. Concentrated NW samples were subsequently diluted 1:10 or 1:25 for IgG and 1:50 or 1:100 for IgA using Diluent 100. AU per ml values were calculated for each sample using relevant MSD reference standard except for anti-XBB.1.16 IgA titers, for which the SARS-CoV-2 WA1 reference standard was used.

### ACE2–S binding inhibition

ACE2 binding inhibition was performed as previously described^36^. Briefly, BAL and NWs were concentrated tenfold with Amicon Ultra centrifugal filters with a 30-kDa MWCO (Millipore). Concentrated samples were subsequently diluted 1:5 in Diluent 100 (MSD). The ACE2 binding inhibition assay was performed with a V-Plex SARS-CoV-2 Panel 32 (ACE2) kit (MSD), as per the manufacturer’s instructions. Plates were read on an MSD Sector S 600 instrument.

For the XBB.1.16 variant, ACE2 binding inhibition was performed using a modified MSD platform assay. Briefly, after blocking MSD Streptavidin Multi-Array 384-well plates with Blocker A (MSD), the plates were coated with 1 μg ml^–1^ biotinylated SARS-CoV-2 XBB.1.16 S-2P and incubated for 1 h at room temperature. The plates were then washed five times with wash buffer (1× PBS containing 0.05% Tween-20). Diluted samples were added to the coated plates and incubated for 1 h at room temperature. MSD Sulfo-Tag human ACE2 protein was diluted 1:200 and added to the plates. After a 1-h incubation at room temperature, the plates were washed five times with wash buffer and read on an MSD Sector S 600 instrument after the addition of Gold Read Buffer B (MSD). The results are reported as percent inhibition.

For depletion of specific antibody classes from concentrated NW and BAL (week 44) samples, we used Pierce Protein G magnetic beads (Thermo Fisher Scientific), according to the manufacturer’s instructions. Briefly, 50 µl of sample was incubated for 2 h at room temperature with 50 µl of Protein G magnetic beads previously equilibrated with PBS (pH 7.4). After incubation, beads were removed by a magnetic stand, and the flow-through was collected as IgG-depleted fluid. The magnetic beads were then washed three times with PBS (pH 7.4) and subsequently incubated with 100 µl of Pierce IgG Elution Buffer (pH 2.0; Thermo Fisher Scientific) for 10 min at room temperature. The beads were removed using a magnetic stand, and the eluant was collected and dialyzed against PBS (pH 7.4) with Slide-A-Lyzer MINI Dialysis Devices (20-kDa MWCO; Thermo Fisher Scientific). The dialyzed eluant was collected as IgA-depleted fluid. Concentrations of IgG and IgA in the complete mucosal fluid, flow-through and eluant were determined by enzyme-linked immunosorbent assay using an Isotyping Panel 1 NHP/Human kit (MSD). An equal concentration of IgG and IgA for each sample was used in ACE2 binding inhibition assays. Comparison of total IgA to secretory IgA was performed using an anti-monkey secretory component primary antibody (polyclonal, Nordic MUbio GAMon/SC; 1 µg ml^–1^).

### Variant S-2P antigen production

The biotinylated SARS-CoV-2 variant S-2P was produced as previously described^100^ with an updated plasmid encoding the sequence of BA.5 or XBB.1.16 S-2P flanked by an HRV 3C cleavable single-chain Fc tag and an Avi tag. Briefly, Avi-tagged S-2P expressed by transient transfection was captured by protein A resin and collected in the flow-through after concurrent HRV 3C cleavage and BirA biotinylation. Afterward, S-2P trimers were purified on a Superdex 200 16/600 gel filtration column (Cytiva) equilibrated with PBS.

### B cell analysis

Cryopreserved PBMCs and BAL cells were thawed, washed briefly with phenol-free RPMI with 4% heat-inactivated newborn calf serum and incubated with Aqua live/dead viability dye (Invitrogen, L34957; 1:800) for 20 min at room temperature. Cells were stained with the following antibodies (monoclonal unless indicated) for 20 min at room temperature: (1) IgD FITC (goat polyclonal, Southern Biotech, 2030-02) at 1:40, (2) IgM PerCP-Cy5.5 (clone G20-127, BD Biosciences, 561285) at 1:40, (3) IgA Dylight 405 (goat polyclonal, Jackson ImmunoResearch, 109-475-011) at 1:40, (4) CD20 BV570 (clone 2H7, Biolegend, 302332) at 1:40, (5) CD27 BV650 (clone O323, Biolegend, 302828) at 1:20, (6) CD14 BV785 (clone M5E2, Biolegend, 301840) at 1:80, (7) CD16 BUV496 (clone 3G8, BD Biosciences, 564653) at 1:40, (8) CD4 BUV737 (clone SK3, BD Biosciences, 564305) at 1:40, (9) CD19 APC (clone J3-119, Beckman Coulter, IM2470U) at 1:20, (10) IgG Alexa 700 (clone G18-145, BD Biosciences, 561296) at 1:20, (11) CD3 APC-Cy7 (clone SP34.2, BD Biosciences, 557757) at 1:40, (12) CD38 PE (clone OKT10, Caprico Biotech, 100826) at 1:640, (13) CD21 PE-Cy5 (clone B-ly4, BD Biosciences, 551064) at 1:20 and (14) CXCR5 PE-Cy7 (clone MU5UBEE, Thermo Fisher, 25-9185-42) at 1:40. Surface-stained cells were then incubated with streptavidin-BV605-labeled (BD Biosciences) WA1 S-2P, streptavidin-BUV661-labeled (BD Biosciences) BA.5 S-2P and streptavidin-BUV395-labeled XBB.1.16 S-2P for 30 min at 4 °C (protected from light). Cells were washed and fixed in 0.5% formaldehyde (Tousimis Research) before data acquisition. All antibodies were titrated on NHP PBMCs to determine the optimal concentration. Samples were acquired on a BD FACSymphony cytometer with BD FACS Diva version 9.3.1 and analyzed for B cell phenotype and Boolean probe binding frequencies using FlowJo version 10.9.0 (BD Biosciences). Microsoft Excel version 16.87 was used for data sorting and background subtraction. Boolean visualization was generated using Spice 6 (Vaccine Research Center)^101^ and Prism 9 (GraphPad Software).

### Intracellular cytokine staining

Intracellular cytokine staining was performed as previously described^35,102^. Briefly, cryopreserved PBMCs and BAL cells were thawed and rested overnight at 37 °C and 5% CO_2_. After resting, cells were stimulated with SARS-CoV-2 S protein (S1 and S2) and N peptide pools (JPT Peptides) at a final concentration of 2 μg ml^–1^ in the presence of 3 mM monensin for 6 h. The S1, S2 and N peptide pools are comprised of 158, 157 and 102 individual peptides, respectively, as 15-mers overlapping by 11 amino acids in 100% DMSO. Negative controls received an equal concentration of DMSO to that of the peptide pools (final concentration of 0.5%). The following monoclonal antibodies were used: (1) CD3 APC-CY7 (clone SP34.2, BD Biosciences, 557757) at 1:640, (2) CD4 PE-CY5.5 (clone SK3, Thermo Fisher, 35-0047-42) at 1:20, (3) CD8 BV570 (clone RPA-T8, Biolegend, 301038) at 1:80, (4) CD45RA PE-CY5 (clone 5H9, BD Biosciences, 552888) at 1:2,500, (5) CCR7 BV650 (clone GO43H7, Biolegend, 353234) at 1:10, (6) CXCR5 PE (clone MU5UBEE, Thermo Fisher, 12-9185-42) at 1:10, (7) CXCR3 BV711 (clone 1C6/CXCR3, BD Biosciences, 563156) at 1:20, (8) PD-1 BUV737 (clone EH12.1, BD Horizon, 612792) at 1:40, (9) ICOS PE-CY7 (clone C398.4A, Biolegend, 313520) at 1:640, (10) CD69 ECD (clone TP1.55.3, Beckman Coulter, 6607110) at 1:40, (11) IFNγ Ax700 (clone B27, Biolegend, 506516) at 1:640, (12) IL-2 BV750 (clone MQ1-17H12, BD Biosciences, 566361) at 1:80, (13) IL-4 BB700 (clone MP4-25D2, BD Biosciences, custom order) at 1:20, (14) TNF FITC (clone Mab11, BD Biosciences, 554512) at 1:80, (15) IL-13 BV421 (clone JES10-5A2, BD Biosciences, 563580) at 1:20, (16) IL-17A BV605 (clone BL168, Biolegend, 512326) at 1:40, (17) IL-21 Ax647 (clone 3A3-N2.1, BD Biosciences, 560493) at 1:10 and (18) CD154 BV785 (clone 24–31, Biolegend, 310842) at 1:20. An Aqua live/dead fixable dead cell stain kit (Invitrogen, L34957; 1:800) was used to exclude dead cells. All antibodies were previously titrated to determine the optimal concentration. Samples were acquired on a BD FACSymphony flow cytometer with BD FACS Diva version 9.3.1, while data sorting and background subtraction were conducted in Microsoft Excel version 16.87. Data were analyzed using FlowJo version 10.9.0 (Treestar).

### Statistical analysis

Humoral, cellular and virus assays were log transformed as appropriate and, where indicated, are reported as geometric means, with error bars depicting geometric standard deviation. For statistical analyses of virus titers in Fig. 1, the three groups given adenoviral-vectored vaccine were compared to the pooled control arms at each time point. For each comparison, Kruskal–Wallis tests were used to compare all groups simultaneously at *α* = 0.05, followed by comparisons for each vaccinated group and the pooled controls using Wilcoxon rank-sum tests at *α* = 0.05/2 if the overall test was statistically significant. Reported pairwise *P* values were doubled to account for this adjustment.

Statistical analyses comparing time points were performed using Wilcoxon signed-rank tests, and comparisons between groups were conducted with Wilcoxon rank-sum tests. Reported correlations and the corresponding *P* values are based on Spearman rank correlations. Values below the LOD were set to the limit, which varied by assay. Values of 0 on variables that were analyzed on a log scale were set to half the minimum of the nonzero data. Because all of these analyses were nonparametric, the results were unaffected by the precise values used.

The only parametric analyses involved fitting linear models to predict sgRNA copy number (log_10_ sgRNA N at day 2) as a function of vaccination group and candidate immune assays. Candidate immune assays were screened one by one (on a log scale as appropriate) to determine if any of the assay readouts were able to predict sgRNA N copy number at day 2 well enough that the vaccination group was no longer significantly predictive after adjusting for the immune readout. Sixteen immune assays were considered for predicting sgRNA N in BAL, and ten assays were considered for predicting sgRNA N in NSs. Because these linear models are parametric and therefore sensitive to the values used for results below the LOD or off the log scale, they were refit with small changes in the assigned values to assess robustness, and the conclusions did not change.

All comparisons and predictive models should be considered hypothesis generating as there were few adjustments for multiple comparisons. Pairwise *P* values are indicated by asterisks in the figures, and the sample *n* is listed in corresponding figure legends. All analyses were conducted using R version 4.3.0 unless otherwise specified.

### Reporting summary

Further information on research design is available in the Nature Portfolio Reporting Summary linked to this article.

## Online content

Any methods, additional references, Nature Portfolio reporting summaries, source data, extended data, supplementary information, acknowledgements, peer review information; details of author contributions and competing interests; and statements of data and code availability are available at 10.1038/s41590-024-01951-5.

## Supplementary information

Supplementary Information Supplementary Tables 1–5 and Fig. 1.

Reporting Summary

## Data Availability

All data are available in the main text, Supplementary Information or Extended Data figures. The Wuhan-Hu-1 SARS-CoV-2 reference is available at GenBank NC_045512. XBB.1.16 and EG.5.1 sequences are available at GISAID EPI_ISL_17417328 and EPI_ISL_17977757, respectively.
