# Supplementary material for: Mucosal adenovirus vaccine boosting elicits IgA and durably prevents XBB.1.16 infection in nonhuman primates
Source: Nat Immunol. 2024 Sep 3;25(10):1913–27. doi: 10.1038/s41590-024-01951-5 (PMC11436372; doi:10.1038/s41590-024-01951-5)
Supplement: Supplementary file 1 — Supplementary Tables 1–5 and Fig. 1. [file 41590_2024_1951_MOESM1_ESM.pdf]

nature immunology

Supplementary information

https://doi.org/10.1038/s41590-024-01951-5

Mucosal adenovirus vaccine boosting elicits IgA and durably prevents XBB.1.16 infection in nonhuman primates

In the format provided by the authors and unedited

Supplementary Data Table of Contents

Supplementary Tables

Table 1. Relative increase in serum titers to XBB.1.16 following challenge …………..…………….…………… 2

Table 2. AE immunization preferentially expands antigen-specific memory B cell compartment in the lungs 3

Table 3. Durable boost in mucosal antibodies following mucosal vaccination ………...……………………..…... 4

Table 4. IgG and IgA contribute to protective airway responses in mucosally vaccinated NHP ….………..….… 7

Table 5. AE immunization preferentially expands antigen-specific memory T cell compartment in the lungs ….. 9

Supplementary Figures

Figure 1. Non-specific T cell recruitment into the lungs following IM, IN or AE immunization …………..…… 11

Group	Fold-increase of serum anti-XBB.1.16 IgG binding titers
mRNA-1273 (IM) x2 + mRNA-1273.222 (IM)	2.06
mRNA-1273 (IM) x2 + Bivalent ChAd (IN)	2.51
mRNA-1273 (IM) x2 + Bivalent ChAd (AE)	0.67
Bivalent ChAd (AE) x 1	4.03

Supplementary Table 1. Relative increase in serum titers to XBB.1.16 following challenge

Sera were collected pre- (week 48) and post- (day 15) XBB.1.16 challenge. Fold-increase in anti-XBB.1.16 IgG GMT in AU / mL described. AU below a value of 1 were replaced with a value of 1 prior to calculation. Related to **Fig. 2g**.

a

S-specific memory B cell frequency<br>Week 8 (post-dose 2) vs. Week 36 (post-boost)				
Group	**BAL**		**PBMC**	
	p	*Fold-change*	*p*	*Fold-change*
IM boost	>0.05	1.07	0.0078	1.72
IN boost	>0.05	2.05	0.0313	0.45
AE boost	0.0313	19.99	>0.05	0.68

b

S-specific memory B cell frequency<br>Week 36 (post-boost) for indicated group in comparison to IM boost group				
Group	**BAL**		**PBMC**	
	p	*Fold-change*	*p*	*Fold-change*
IM boost	-	-	-	-
IN boost	>0.05	0.76	0.0007	0.22
AE boost	0.0007	20.37	0.0007	0.25
AE prime	0.0040	13.31	0.0040	0.28

Supplementary Table 2. AE immunization preferentially expands antigen-specific memory B cell compartment in the lungs

(**a**) Two-sided Wilcoxon signed-rank tests conducted for S-specific memory B cell frequencies at week 36 (post-boost) compared to week 8 (post-dose 2) per group, and (**b**) two-sided Wilcoxon rank-sum tests conducted for each indicated vaccinated cohort in comparison to IM boosted benchmark at week 36 (post-boost). *p* values and fold-changes in geometric mean frequency indicated. S-specific frequencies of 0% were set to half the minimum of the non-zero data prior to calculation of fold-change. Statistically significant increased B cell frequencies are indicated in red while significant decreases are indicated in orange. Related to **Fig. 3**.

a

BAL IgG									
Group		**IM boost**		**IN boost**		**AE boost**		**AE prime**	
Comparison		p value	Fold-change	p value	Fold-change	p value	Fold-change	p value	Fold-change
WA1	Post-boost Wk 34 vs 28	0.0078	376.00	0.0313	89.51	0.0313	361.12	-	-
	Prechallenge Wk 48 vs 34	0.0078	0.20	>0.05	0.66	>0.05	0.70	>0.05	7.45
BA.5	Post-boost Wk 34 vs 28	0.0078	124.55	0.0313	25.45	0.0313	103.60	-	-
	Prechallenge Wk 48 vs 34	0.0078	0.23	>0.05	0.57	>0.05	0.73	>0.05	24.85
XBB.1.16	Post-boost Wk 34 vs 28	0.0078	88.25	0.0313	19.45	0.0313	87.08	-	-
	Prechallenge Wk 48 vs 34	0.0078	0.22	>0.05	0.56	>0.05	0.65	>0.05	19.77

b

BAL IgA									
Group		**IM boost**		**IN boost**		**AE boost**		**AE prime**	
Comparison		p value	Fold-change	p value	Fold-change	p value	Fold-change	p value	Fold-change
WA1	Post-boost Wk 34 vs 28	>0.05	2.64	0.0313	28.83	0.0313	1323.51	-	-
	Prechallenge Wk 48 vs 34	>0.05	0.58	>0.05	0.96	>0.05	1.04	>0.05	2.23
BA.5	Post-boost Wk 34 vs 28	0.0078	1.89	0.0313	13.00	0.0313	537.50	-	-
	Prechallenge Wk 48 vs 34	>0.05	0.67	>0.05	0.75	>0.05	1.44	>0.05	10.80
XBB.1.16	Post-boost Wk 34 vs 28	>0.05	1.00	0.0313	7.50	0.0313	346.74	-	-
	Prechallenge Wk 48 vs 34	>0.05	1.06	>0.05	0.89	>0.05	1.53	>0.05	12.62

c

NW IgG									
	Group	IM boost		IN boost		AE boost		AE prime	
	Comparison	p value	Fold-change	p value	Fold-change	p value	Fold-change	p value	Fold-change
WA1	Post-boost Wk 34 vs 28	0.0391	6.96	>0.05	1.90	>0.05	1.10	-	-
	Prechallenge Wk 48 vs 34	>0.05	0.51	>0.05	2.21	>0.05	3.11	>0.05	14.26
BA.5	Post-boost Wk 34 vs 28	0.0156	9.24	>0.05	1.99	>0.05	1.20	-	-
	Prechallenge Wk 48 vs 34	>0.05	0.44	>0.05	2.03	>0.05	2.91	>0.05	87.22
XBB.1.16	Post-boost Wk 34 vs 28	0.0391	4.58	>0.05	1.89	>0.05	1.12	-	-
	Prechallenge Wk 48 vs 34	>0.05	0.38	>0.05	1.95	>0.05	2.45	>0.05	24.94

d

NW IgA									
	Group	IM boost		IN boost		AE boost		AE prime	
	Comparison	p value	Fold-change	p value	Fold-change	p value	Fold-change	p value	Fold-change
WA1	Post-boost Wk 34 vs 28	>0.05	2.26	>0.05	12.63	>0.05	4.24	-	-
	Prechallenge Wk 48 vs 34	>0.05	0.37	0.0313	4.09	>0.05	1.34	>0.05	4.29
BA.5	Post-boost Wk 34 vs 28	>0.05	2.02	>0.05	10.97	>0.05	3.85	-	-
	Prechallenge Wk 48 vs 34	0.0078	0.25	0.0313	4.15	>0.05	1.51	>0.05	13.78
XBB.1.16	Post-boost Wk 34 vs 28	>0.05	1.87	>0.05	9.41	>0.05	2.99	-	-
	Prechallenge Wk 48 vs 34	0.0781	0.27	0.0313	4.27	>0.05	1.36	>0.05	2.48

Supplementary Table 3. Durable boost in mucosal antibodies following mucosal vaccination

BAL (**a-b**) and NW (**c-d**) were collected pre-boost (week 28), post-boost (week 34) and pre-challenge (week 48). IgG (**a, c**) and IgA (**b, d**) binding titers in AU / mL measured to WA1, BA.5 or XBB.1.16 S as indicated. AU below a value of 1 were replaced with a value of 1 before

calculation of fold-change in anti-S antibody GMT. AE prime cohort received no vaccine prior to the initial regimen at week 32 and thus responses were not measured at week 28. Note that pre-boost data is derived per individual group and is used for determination of fold-changes whereas fold-changes in the text reflect the combined values across all mRNA-primed cohorts prior to boost. Comparisons between timepoints were done using two-sided Wilcoxon signed-rank tests. Statistically significant increases in antibody titers are indicated in red while significant contractions are indicated in orange. Related to **Fig. 4**.

a

BAL ACE2 – variant S binding inhibition									
	Group	IM boost		IN boost		AE boost		AE prime	
	Comparison	p value	Fold-change	p value	Fold-change	p value	Fold-change	p value	Fold-change
WA1	Post-boost Wk 34 vs 28	0.0078	8.53	>0.05	1.71	0.0313	13.93	-	-
	Prechallenge Wk 48 vs 34	0.0078	0.30	>0.05	0.42	>0.05	0.94	>0.05	0.56
BA.5	Post-boost Wk 34 vs 28	>0.05	3.54	N.D.	1.00	>0.05	32.80	-	-
	Prechallenge Wk 48 vs 34	>0.05	0.28	N.D.	1.00	>0.05	0.89	>0.05	5.63
XBB.1.16	Post-boost Wk 34 vs 28	>0.05	0.70	0.0313	5.07	>0.05	2.35	-	-
	Prechallenge Wk 48 vs 34	>0.05	0.13	0.0313	2.37	>0.05	0.98	>0.05	0.62

b

NW ACE2 – variant S binding inhibition									
	Group	IM boost		IN boost		AE boost		AE prime	
	Comparison	p value	Fold-change	p value	Fold-change	p value	Fold-change	p value	Fold-change
WA1	Post-boost Wk 34 vs 28	>0.05	1.25	>0.05	0.65	>0.05	0.65	-	-
	Prechallenge Wk 48 vs 34	>0.05	0.85	0.0313	3.40	>0.05	2.18	>0.05	0.24

c

BAL immunoglobulin class depletion									
	Group	IM boost		IN boost		AE boost		AE prime	
	Comparison	p value	Fold-change	p value	Fold-change	p value	Fold-change	p value	Fold-change
IgG Depletion	WA1	0.0078	0.11	>0.05	0.93	>0.05	0.37	>0.05	1.16
	BA.5	>0.05	0.54	>0.05	0.84	>0.05	0.49	>0.05	0.62
	XBB.1.16	0.0078	0.21	N.D.	1.00	0.0313	0.21	>0.05	0.86
IgA Depletion	WA1	>0.05	1.48	>0.05	1.85	>0.05	0.87	>0.05	0.57
	BA.5	>0.05	1.68	>0.05	0.88	>0.05	0.56	>0.05	1.19
	XBB.1.16	0.0391	0.57	N.D.	1.00	0.0313	0.37	>0.05	0.90

d

NW immunoglobulin class depletion									
	Group	**IM boost**		**IN boost**		**AE boost**		**AE prime**	
	Comparison	p value	Fold-change	p value	Fold-change	p value	Fold-change	p value	Fold-change
IgG Depletion	WA1	0.0156	0.32	>0.05	0.93	0.0313	0.62	>0.05	1.24
IgA Depletion	WA1	>0.05	0.74	0.0313	0.13	>0.05	0.55	>0.05	0.69

Supplementary Table 4. IgG and IgA contribute to protective airway responses in mucosally vaccinated NHP

BAL (**a, c**) and NW (**b, d**) were collected pre-boost (week 28), post-boost (week 34) and pre-challenge (week 48) for longitudinal analysis in **a-b** and at a single late memory timepoint (week 44) for immunoglobulin class depletion in **c-d**. WA1, BA.5 and XBB.1.16 S binding to ACE2 was measured with and without the addition of mucosal fluids to determine percentage inhibition as a surrogate for neutralizing antibodies. (**a-b**) Fold-changes in median binding inhibition shown. Note that pre-boost data is derived per individual group and is used for determination of fold-changes whereas fold-changes in the text reflect the combined values across all mRNA-primed cohorts prior to boost. (**c-d**) Fold-changes in median binding inhibition shown for complete fluid and depleted fluid. Any group with median inhibition below 1% was given a value of 1% before calculation of fold-change. AE prime cohort received no vaccine prior to the initial regimen at week 32 and thus fold-changes between pre- and post-boost timepoints are not reported. N.D. or "Not Determined" refers to samples for which there were insufficient differences (less than 3 unique values) between timepoints. Comparisons between timepoints were done using two-sided Wilcoxon signed-rank tests. Statistically significant increases in inhibitory responses are indicated in red while significant reductions are indicated in orange. Related to **Fig. 5**.

a

**S-specific T_H1 frequency**								
Tissue	**PBMC**				**BAL**			
Group	**IM boost**	**IN boost**	**AE boost**	**AE prime**	**IM boost**	**IN boost**	**AE boost**	**AE prime**
Comparison	Fold-change				Fold-change			
Post-boost Wk 34 vs 6	0.96	0.74	1.09	-	0.56	1.59	12.81	-
Pre-challenge Wk 48 vs 34	0.89	1.00	1.19	1.00	1.21	0.74	1.97	1.06
Post-challenge Day 4 vs Wk 48	0.95	1.00	0.92	N.D.	1.07	1.30	0.65	0.36
Post-challenge Day 15 vs Wk 48	1.24	1.06	0.85	1.66	1.73	5.66	0.90	1.58

b

**S-specific $CD8^+$ T cell frequency**								
Tissue	**PBMC**				**BAL**			
Group	**IM boost**	**IN boost**	**AE boost**	**AE prime**	**IM boost**	**IN boost**	**AE boost**	**AE prime**
Comparison	Fold-change				Fold-change			
Post-boost Wk 34 vs 6	0.83	1.00	1.13	-	0.95	1.11	7.75	-
Pre-challenge Wk 48 vs 34	0.93	1.00	1.53	1.40	0.63	0.96	9.27	2.03
Post-challenge Day 4 vs Wk 48	1.48	1.00	0.75	N.D.	1.18	1.11	0.70	0.40
Post-challenge Day 15 vs Wk 48	1.41	1.02	0.76	0.86	2.53	4.44	0.73	0.54

c

**S-specific T_H2 and T_{FH} frequencies**								
Tissue	**PBMC – T_H2**				**PBMC - T_{FH}**			
Group	**IM boost**	**IN boost**	**AE boost**	**AE prime**	**IM boost**	**IN boost**	**AE boost**	**AE prime**
Comparison	Fold-change				Fold-change			
Post-boost Wk 34 vs 6	1.00	1.00	1.00	-	0.72	0.40	1.43	-
Pre-challenge Wk 48 vs 34	1.00	1.00	1.00	1.00	0.46	0.63	0.85	0.60

Post-challenge Day 4 vs Wk 48	1.00	1.00	1.00	N.D.	0.62	0.69	0.46	N.D.
Post-challenge Day 15 vs Wk 48	1.00	1.00	1.00	1.00	1.83	2.32	0.57	1.04

Supplementary Table 5. AE immunization preferentially expands antigen-specific memory T cell compartment in the lungs

PBMC and BAL fluid were collected at weeks 6 (post-prime), 34 (post-boost) and 48 (pre-challenge) as well as on days 4 and 15 post-challenge. Lymphocytes were stimulated with SARS-CoV-2 S1 and S2 peptide pools (WA1) and then measured by intracellular staining. (**a, c**) Fold-changes for indicated timepoints in percentage of memory $CD4^+$ T cells with (**a**) T_H1 markers (IL-2, TNF or IFNγ), (**c**) T_H2 markers (IL-4 or IL-13) or T_{FH} cells expressing CD40L following stimulation. (**b**) Fold-changes for indicated timepoints in percentage of memory $CD8^+$ T cells expressing IL-2, TNF or IFNγ following stimulation. Frequencies below 0.1% were set to 0.1% prior to calculation of fold-change in geometric mean S-specific frequency. AE prime cohort received no vaccine prior to the initial regimen at week 32 and thus fold-changes between pre- and post-boost timepoints are not reported. N.D. or "Not Determined" refers to comparisons in which there were less than 2 samples contributing to calculation of group frequency for one of the timepoints. Related to **Fig. 6**.

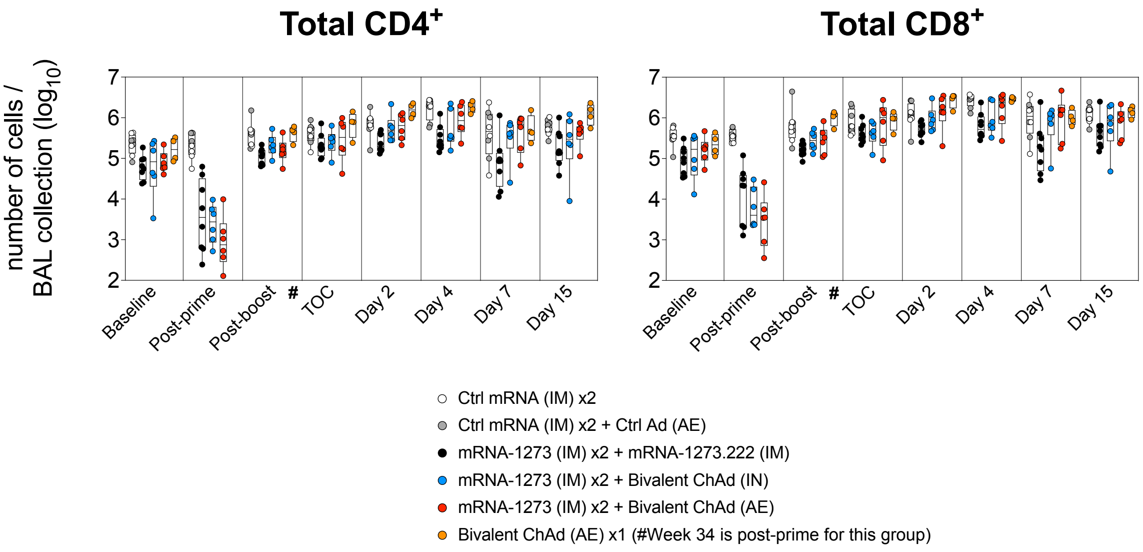

Supplementary Figure 1. Non-specific T cell recruitment into the lungs following IM, IN or AE immunization

BAL fluid was collected pre-vaccination (baseline) and at weeks 6 (post-prime), 34 (post-boost) and 48 (time of challenge, TOC) as well as on days 2, 4, 7 and 15 post-challenge. Absolute number of total $CD4^+$ and $CD8^+$ T cells (memory and naïve) determined by flow cytometry. Circles, boxes and horizontal lines represent individual animals, interquartile range and median, respectively, while minima and maxima are denoted at whisker termini. # indicates that while sample collection for AE prime cohort (orange) occurred on week 34, week 34 was two weeks following the single AE prime rather than two weeks following a boost as in all other groups. Immunizations include control mRNA (ctrl mRNA) via IM route, control adenovirus vector (ctrl Ad) via AE route, mRNA-1273 via IM route, mRNA-1273.222 via IM route, and bivalent ChAd-SARS-CoV-2-S via AE or IN route. Number of NHP per group (n) are as follows: control = 8; IM boost = 8; IN boost = 6; AE boost = 6; AE prime = 4.
