# Supplementary material for: Mucosal adenovirus vaccine boosting elicits IgA and durably prevents XBB.1.16 infection in nonhuman primates
Source: Nat Immunol. 2024 Sep 3;25(10):1913–27. doi: 10.1038/s41590-024-01951-5 (PMC11436372; doi:10.1038/s41590-024-01951-5)
Supplement: Supplementary file 2 — Reporting Summary [file 41590_2024_1951_MOESM2_ESM.pdf]

nature portfolio

Corresponding author(s): Robert A. Seder and Daniel C. Douek

Last updated by author(s): Aug 4, 2024

# Reporting Summary

Nature Portfolio wishes to improve the reproducibility of the work that we publish. This form provides structure for consistency and transparency in reporting. For further information on Nature Portfolio policies, see our Editorial Policies and the Editorial Policy Checklist.

## Statistics

For all statistical analyses, confirm that the following items are present in the figure legend, table legend, main text, or Methods section.

| n/a | Confirmed | |
|---|---|---|
| ☐ | ☒ | The exact sample size (*n*) for each experimental group/condition, given as a discrete number and unit of measurement |
| ☐ | ☒ | A statement on whether measurements were taken from distinct samples or whether the same sample was measured repeatedly |
| ☐ | ☒ | The statistical test(s) used AND whether they are one- or two-sided<br>*Only common tests should be described solely by name; describe more complex techniques in the Methods section.* |
| ☐ | ☒ | A description of all covariates tested |
| ☐ | ☒ | A description of any assumptions or corrections, such as tests of normality and adjustment for multiple comparisons |
| ☐ | ☒ | A full description of the statistical parameters including central tendency (e.g. means) or other basic estimates (e.g. regression coefficient) AND variation (e.g. standard deviation) or associated estimates of uncertainty (e.g. confidence intervals) |
| ☐ | ☒ | For null hypothesis testing, the test statistic (e.g. *F*, *t*, *r*) with confidence intervals, effect sizes, degrees of freedom and *P* value noted<br>*Give P values as exact values whenever suitable.* |
| ☒ | ☐ | For Bayesian analysis, information on the choice of priors and Markov chain Monte Carlo settings |
| ☒ | ☐ | For hierarchical and complex designs, identification of the appropriate level for tests and full reporting of outcomes |
| ☐ | ☒ | Estimates of effect sizes (e.g. Cohen's *d*, Pearson's *r*), indicating how they were calculated |

*Our web collection on statistics for biologists contains articles on many of the points above.*

## Software and code

Policy information about availability of computer code

| | |
|---|---|
| Data collection | All flow cytometry data was collected in BD FACS Diva version 9.3.1. |
| Data analysis | All statistical analyses were done using R version 4.3.0. XBB.1.16 challenge stock sequence reads were analyzed in CLC Genomics Workbench version 23.0.1. Pseudovirus and live virus neutralization titers were interpolated using a NAB analysis module on Labkey web-based server and GraphPad Prism version 9.2.0, respectively. Pseudovirus neutralization assays were performed on integrated automation platforms consisting of a Biomek liquid handler from Beckman Coulter operated through Beckman Coulter SAMI EX software version 5.0. Flow cytometry samples were acquired on a BD FACSymphony cytometer with BD FACS Diva version 9.3.1. Microsoft Excel version 16.87 was used for data sorting and background subtraction. Boolean visualization of B cell data was generated using Spice 6 (Vaccine Research Center, Bethesda, MD) and GraphPad Prism version 9. All other flow cytometry data analyzed in BD FlowJo version 10.9.0. |

For manuscripts utilizing custom algorithms or software that are central to the research but not yet described in published literature, software must be made available to editors and reviewers. We strongly encourage code deposition in a community repository (e.g. GitHub). See the Nature Portfolio guidelines for submitting code & software for further information.

## Data

Policy information about availability of data

All manuscripts must include a data availability statement. This statement should provide the following information, where applicable:

- Accession codes, unique identifiers, or web links for publicly available datasets
- A description of any restrictions on data availability
- For clinical datasets or third party data, please ensure that the statement adheres to our policy

All data are available in the main text, the supplementary tables or in the Extended Data figures. Wuhan-Hu-1 SARS-CoV-2 reference is available at GenBank no. NC_045512. XBB.1.16 and EG.5.1 sequences are available at GISAID no. EPI_ISL_17417328 and EPI_ISL_17977757, respectively.

## Research involving human participants, their data, or biological material

Policy information about studies with human participants or human data. See also policy information about sex, gender (identity/presentation), and sexual orientation and race, ethnicity and racism.

| | |
|---|---|
| Reporting on sex and gender | No human research participants used in this study. |
| Reporting on race, ethnicity, or other socially relevant groupings | Not applicable |
| Population characteristics | Not applicable |
| Recruitment | Not applicable |
| Ethics oversight | Not applicable |

Note that full information on the approval of the study protocol must also be provided in the manuscript.

# Field-specific reporting

Please select the one below that is the best fit for your research. If you are not sure, read the appropriate sections before making your selection.

☒ Life sciences ☐ Behavioural & social sciences ☐ Ecological, evolutionary & environmental sciences

For a reference copy of the document with all sections, see nature.com/documents/nr-reporting-summary-flat.pdf

# Life sciences study design

All studies must disclose on these points even when the disclosure is negative.

**Sample size**

In this study, we aimed to assess protection from SARS-CoV-2 challenge after boosting with mucosally-delivered ChAd-SARS-CoV-2-S (n=6/group) or intramuscularly-delivered mRNA-1273.222 (n=8). After SARS-CoV-2 challenge, the amount of virus RNA detectable in nasal swabs and bronchoalveolar lavage would be evaluated in all groups. Our primary comparison was protection elicited by any of the vaccinated groups in relation to the untreated control groups. These control groups were composed of a cohort of NHP that received control mRNA (n=4) or control mRNA followed by control ChAd (n=4), which we planned to combine into a single control group for statistical analyses if no differences in virus titers were observed between the cohorts. Our secondary objective was to compare protection provided by any of the mucosally-boosted groups with the intramuscularly-boosted benchmark group. We estimated the standard deviation in virus loads to be approximately 0.5 logs based on historical data from other NHP studies. This number of animals (n=6-8/group) thus allowed us 80% power to detect differences in antibody titers that differ by 0.75 logs; this is sufficient for our study, in which we hypothesized a decrease of over 1.0 log. Finally, to investigate the potential of a mucosally-delivered virus-vectored vaccine in an unexposed population and allow a comparison between immune responses following boosting versus primary immunization, we also administered a single dose of the same ChAd-SARS-CoV-2-S vaccine to naïve NHP via aerosol. This naive cohort was composed of 4 NHP due to the limitations of our already large study and the number of available primates.

**Data exclusions**

No data was excluded from this study.

**Replication**

We used multiple experimental approaches to verify reproducibility of data including both sgRNA and TCID50 analysis for determination of virus replication; upper airway virus load was confirmed through measurement of culturable virus using both nasal swabs and nasal washes. Neutralizing responses were verified using both pseudovirus and authentic virus. We also measured antigen-specific T cell responses using lymphocytes isolated from distinct compartments (blood & BAL) and measured humoral responses using antibodies from multiple compartments (blood, BAL, NW) and multiple antigens (WA1, BA.5 and XBB.1.16 spikes) and found consistent trends.

Additionally we measured virus RNA transcripts by performing two independent PCR assays (for different virus-specific targets). Although the amplified subgenomic transcript (E vs N) was unique to each PCR run (with differences in RNA copy number of ~1log in our prior experiments), and thus the virus RNA copy numbers in this experiment were also different between each PCR run (by ~1log), the kinetics and relative differences between the various cohorts were nearly identical post-challenge between both assays.

Due to the use of these orthogonal approaches to validate our findings, most assays were run a single time. However, the following assays were repeated:
(1) BA.5 pseudovirus neutralizing titers were measured three times by two separate labs. For one run, the BA.5 pseudovirus stock was not produced at the correct concentration, resulting in high neutralizing responses for all groups including the negative controls. For the other two repeats, both labs independently confirmed the same trends and outliers within and across groups. Neutralization assays for all other variants were run a single time.
(2) Antibody binding titers and ACE2 binding inhibition at pre-challenge timepoints were measured at least 2 times via ELISA using either Meso Scale Discovery (MSD)'s Panel 32 or 36, and results were successfully replicated.
(3) ACE2 binding inhibition after selective depletion of IgG or IgA was performed 3x for nasal wash samples with consistent results.
(4) ACE2 binding inhibition after selective depletion of IgA was performed 2x for BAL samples using two unique methods for removal of IgA. Overall results were reproducible although we observed some differences in background and larger effects resulting from IgA depletion when performing the approach which was not used in the manuscript. Importantly, we did not observe any contrasting results from those reported in the manuscript.

Randomization

Animals were stratified into groups for vaccine boosting based on age and weight.

Blinding

Subgenomic RNA analysis, which was the primary endpoint, was conducted by scientists blinded to group status. For all other assays, scientists were not formally blinded as there was high demand for certain specimen / group samples at specific timepoints, requiring judicious use for some of the samples. However, in practice, group status was largely unknown to investigators during performance of experiments.

# Reporting for specific materials, systems and methods

We require information from authors about some types of materials, experimental systems and methods used in many studies. Here, indicate whether each material, system or method listed is relevant to your study. If you are not sure if a list item applies to your research, read the appropriate section before selecting a response.

## Materials & experimental systems

| n/a | Involved in the study | |
|---|---|---|
| ☐ | ☒ | Antibodies |
| ☐ | ☒ | Eukaryotic cell lines |
| ☒ | ☐ | Palaeontology and archaeology |
| ☐ | ☒ | Animals and other organisms |
| ☒ | ☐ | Clinical data |
| ☒ | ☐ | Dual use research of concern |
| ☒ | ☐ | Plants |

## Methods

| n/a | Involved in the study | |
|---|---|---|
| ☒ | ☐ | ChIP-seq |
| ☐ | ☒ | Flow cytometry |
| ☒ | ☐ | MRI-based neuroimaging |

## Antibodies

Antibodies used

We used the following antibodies for ICS:
1. Live/dead fixable aqua dead cell stain, Invitrogen #L34957 – Lot #2420618 (1:800)
2. CD45RA PE-CY5, clone 5H9, BD Biosciences #552888 – Lot #7144708 (1:2500)
3. CD4 PE-CY5.5, clone SK3, Thermo Fisher #35-0047-42 – Lot #2516573 (1:20)
4. ICOS PE-CY7, clone C398.4A, Biolegend #313520 – Lot #B293719 (1:640)
5. CD8 BV570, clone RPA-T8, Biolegend #301038 – Lot #B346256 (1:80)
6. CCR7 BV650, clone GO43H7, Biolegend #353234 – Lot #B340645 (1:10)
7. CXCR3 BV711, clone 1C6/CXCR3, BD Biosciences #563156 – Lot #2129036 (1:20)
8. PD-1 BUV737, clone EH12.1, BD Horizon #612792 – Lot #0206107, 0303349, 2059784 (1:40)
9. TNF FITC, clone Mab11, BD Biosciences #554512 – Lot #1145433 (1:80)
10. IL-4 BB700, clone MP4-25D2, BD Biosciences custom order – Lot #1145122 (1:20)
11. CXCR5 PE, clone MU5UBEE, Thermo Fisher #12-9185-42 – Lot #2404260 (1:10)
12. CD69 ECD, clone TP1.55.3, Beckman Coulter #6607110 – Lot #7620070, 7620044, 7620076, 7620097, 7620090 (1:40)
13. IL-21 Ax647, clone 3A3-N2.1, BD Biosciences #560493 – Lot #0225901 (1:10)
14. IFN-g Ax700, clone B27, Biolegend #506516 – Lot #B320892 (1:640)
15. CD3 APC-CY7, clone SP34.2, BD Biosciences #557757 – Lot #0223215 (1:640)
16. IL-13 BV421, clone JES10-5A2, BD Biosciences #563580 – Lot #2265146 (1:20)
17. IL-17A BV605, clone BL168, Biolegend #512326 – Lot #B338018 (1:40)
18. CD154 BV785, clone 24-31, Biolegend #310842 – Lot #B329207 (1:20)
19. IL-2 BV750, clone MQ1-17H12, BD Biosciences #566361 – Lot #8305632, 1137097 (1:80)

We used the following antibodies for B cell analysis:
1. Live/dead fixable aqua dead cell stain, Invitrogen #L34957 – Lot #2420618 (1:800)
2. IgD FITC, goat pAb, Southern Biotech #2030-02 – Lot #A2118-WF09C (1:40)
3. IgM PerCP-Cy5.5, clone G20-127, BD Biosciences #561285 – Lot #0307134 (1:40)
4. IgA Dy405, goat pAb, Jackson ImmunoResearch #109-475-011 – Lot #150866 (1:40)
5. CD20 BV570, clone 2H7, Biolegend #302332 – Lot #B301458 (1:40)
6. CD27 BV650, clone O323, Biolegend #302828 – Lot #B350350 (1:20)
7. CD14 BV785, clone M5E2, Biolegend #301840 – Lot #B327948 (1:80)
8. CD16 BUV496, clone 3G8, BD Biosciences #564653 – Lot #0155949 (1:40)

9. CD4 BUV737, clone SK3, BD Biosciences #564305 – Lot #0282762 (1:40)
10. CD19 APC, clone J3-119, Beckman Coulter #IM2470U – Lot #200093 (1:20)
11. IgG Ax700, clone G18-145, BD Biosciences #561296 – Lot #0135021 (1:20)
12. CD3 APC-Cy7, clone SP34.2, BD Biosciences #557757 – Lot #1152687 (1:40)
13. CD38 PE, clone OKT10, Caprico Biotech #100826 – Lot #8AE4 (1:640)
14. CD21 PE-Cy5, clone B-ly4, BD Biosciences #551064 – Lot #0072939 (1:20)
15. CXCR5 PE-Cy7, clone MU5UBEE, Thermo Fisher #25-9185-42 – Lot #2442267 (1:40)

We used the following antibody for live virus neutralization:
1. SARS-CoV-2 spike protein human IgG1 mAb Alexa R 647, clone CR3022, Cell Signaling #37475 – Lot #5 (1:5000)

We used the following antibody for secretory IgA analysis:
1. Anti-monkey secretory component, goat pAb, Nordic MUbio #GAMon/SC – Lot #6746 (1 µg/mL)

Validation

Validation for ICS assay is described previously {Donaldson, M.M., Kao SF, Foulds KE. OMIP-052: An 18-Color Panel for Measuring Th1, Th2, Th17, and Tfh Responses in Rhesus Macaques. Cytometry A 95, 261-263 (2019)}. All antibodies are advertised to work against human except CXCR5 (clone MU5UBEE) which is raised against Rhesus Macaques.

All antibodies for B- and T-cell assays are titrated, per lot, on PBMC from rhesus macaques for optimal staining condition and then in the context of the full panel for optimization.

Antibody used for measurement of virus for live virus neutralization was previously described in the following publications as listed on the manufacturer's website:
1. Suzuki, Y. et al. Design and lyophilization of lipid nanoparticles for mRNA vaccine and its robust immune response in mice and nonhuman primates. Mol. Ther. Nucleic Acids 30, 226-240 (2022).
2. Barber, K.W., Shrock, E. & Elledge, S.J. CasPlay provides a gRNA-barcoded CRISPR-based display platform for antibody repertoire profiling. Cell Rep. Methods 2, 100318 (2022).

Regarding the antibody used for assessment of secretory IgA, manufacturer website states that "purified free secretory component isolated from pooled rhesus monkey milk is used for immunization... Precipitation reactions have been observed with free and bound secretory component in serum of other old-world Monkeys, including Cercopithecus, Cynomolgus and Baboon. The antiserum may also react with other species as has been observed for Chimpanzee... Tested in immunoelectrophoresis, double radial immunodiffusion and ELISA against a panel of appropriate secretions and purified Ig isotypes. The antiserum reacts with both bound secretory component (secretory IgA) and with the free SC present in monkey secretions. In immunoelectrophoresis against monkey milk, using a high electroendosmosis agar plate, free SC is precipitated in the alph-2 region. The antiserum does not react with other molecular forms of IgA, or with any other secretory or plasma protein."

## Eukaryotic cell lines

Policy information about cell lines and Sex and Gender in Research

Cell line source(s)

For propagation of XBB.1.16 challenge stock and live virus neutralization assays, VeroE6-TMPRSS2 cells were used. Vero-ACE2/TMPRSS2 cells were used for TCID50 assays. Both cell lines were obtained from the Vaccine Research Center, NIH. Vero cells are assumed to have been isolated from a female African green monkey and are the standard cell line used for SARS-CoV-2 virus culture and neutralization assays.

293T-human ACE2 cells (obtained from Drs. Michael Farzan and Huihui Mu at UF Scripps Institute) were used for pseudovirus neutralization assays. Cell lines used for ChAd production include T-REx™-293 Cell Line (Invitrogen, R710-07) for rescue of ChAd-SARS-CoV-2-BA.5-S genome and HEK-293 cells (ATCC, CRL-1573) for scaling up of ChAd vectors. These related cell lines all derive from HEK-293 cells, which were produced when an embryonic kidney cell culture (female) was transformed by sheared adenovirus 5 DNA.

Authentication

Vero cell lines were authenticated by characterization of TMPRSS2 via use of an anti-TMPRSS2 flow antibody and validation of positive ACE2 transduction via staining with SARS-CoV-2 RBD probe, which binds ACE2-expressing cells. ACE2 expression was also validated in the 293T-human ACE2 cell line by staining with anti-human ACE2 antibody.

T-REx™-293 and HEK-293 cells were not authenticated.

Mycoplasma contamination

The cell lines used for virus neutralization, both pseudovirus and live virus, were found to be negative for mycoplasma contamination.

Commonly misidentified lines
(See ICLAC register)

No commonly misidentified cell lines used in this study.

## Animals and other research organisms

Policy information about studies involving animals; ARRIVE guidelines recommended for reporting animal research, and Sex and Gender in Research

Laboratory animals

For this study, we enrolled two- to six-year-old male Indian-origin rhesus macaques (Macaca mulatta).

Wild animals

This study did not involve wild animals.

| | |
|---|---|
| Reporting on sex | All primates were male. We only analyzed male primates as disease severity is more pronounced in male populations (for both humans and animal models) and thus would provide a higher threshold for determination of any protective effect arising from mucosal ChAd-SARS-CoV-2-S. |
| Field-collected samples | No samples were collected from the field. |
| Ethics oversight | All experiments were conducted according to National Institutes of Health (NIH) standards on the humane care and use of laboratory animals, and all procedures approved by and conducted in accordance with regulations of the Animal Care and Use Committees of the NIH Vaccine Research Center (VRC) and BIOQUAL, Inc. (Rockville, Maryland). Animals were housed and cared for in accordance with local, state, federal and institute policies in facilities accredited by the American Association for Accreditation of Laboratory Animal Care (AAALAC), under standards established in the Animal Welfare Act and the Guide for the Care and Use of Laboratory Animals. Animals were housed in ABSL-2 conditions for the immunization phase of the study. Up to a week prior to (for acclimation) and during the challenge phase of the study, animals were housed in ABSL-3 conditions, per Bioqual facility standard operating procedures. |

Note that full information on the approval of the study protocol must also be provided in the manuscript.

# Plants

| | |
|---|---|
| Seed stocks | This study did not involve plants. |
| Novel plant genotypes | *Describe the methods by which all novel plant genotypes were produced. This includes those generated by transgenic approaches, gene editing, chemical/radiation-based mutagenesis and hybridization. For transgenic lines, describe the transformation method, the number of independent lines analyzed and the generation upon which experiments were performed. For gene-edited lines, describe the editor used, the endogenous sequence targeted for editing, the targeting guide RNA sequence (if applicable) and how the editor was applied.* |
| Authentication | *Describe any authentication procedures for each seed stock used or novel genotype generated. Describe any experiments used to assess the effect of a mutation and, where applicable, how potential secondary effects (e.g. second site T-DNA insertions, mosiacism, off-target gene editing) were examined.* |

# Flow Cytometry

## Plots

Confirm that:

☒ The axis labels state the marker and fluorochrome used (e.g. CD4-FITC).

☒ The axis scales are clearly visible. Include numbers along axes only for bottom left plot of group (a 'group' is an analysis of identical markers).

☒ All plots are contour plots with outliers or pseudocolor plots.

☒ A numerical value for number of cells or percentage (with statistics) is provided.

## Methodology

| | |
|---|---|
| Sample preparation | All tissue are rhesus macaque in origin. BAL cells were obtained by PBS lavage under anesthesia. Cells were quantified using Nexcelom Cellaca cell counter, cryopreserved in 90% FBS, 10% DMSO, using a controlled rate freezer (Thermo Fisher Scientific). |
| Instrument | BD FACSymphony X-50 (LSR Fortessa), Model Number N/A – Special Order Research Product. REF 660964 |
| Software | We used BD FACS Diva version 9.3.1 for acquisition. We performed analysis and data export in BD FlowJo 10 (version 10.9.0). Microsoft Excel version 16.87 was used for data sorting and background subtraction while Graphpad Prism 9 was used for graphical figures. Boolean visualization was generated using Spice 6 (Vaccine Research Center, Bethesda, MD) and Prism 9. |
| Cell population abundance | No sorting was performed, but for PBMC T-cell assays, 35% viability threshold was applied for exclusion criteria. For BAL T cell assay, samples with less than 5,000 live CD3 were excluded. No exclusion criteria was specified for B cell assay. |
| Gating strategy | Boundaries between positive and negative staining cell populations were defined based on stained negative control samples. Specifically, positive populations were defined by stimulated (using spike or nucleoprotein peptides) vs. unstimulated (DMSO) conditions (for T-cell assay) or pre-exposure vs. post-challenge conditions (for B-cell assay). Details on gating strategy are listed below.<br><br>For B cell assay: Cells were gated as singlets and live cells on forward and side scatter and a live/dead aqua blue stain. Cells were further gated based on lack of expression of CD3, CD4, CD14 and CD16. B cells were then defined based on expression of CD20 and CD19 whereas memory B cells were gated based on lack of IgD or IgM expression. Finally variant S-2P probe pairs were used to define binding specificity.<br><br>For ICS: Cells were gated as singlets and live cells on forward and side scatter and a live/dead aqua blue stain. CD3+ events were gated as CD4+ or CD8+ T cells. Total memory CD8+ T cells were selected based on expression of CCR7 and CD45RA. Finally, SARS-CoV-2 S-specific memory CD8+ T cells were gated according to co-expression of CD69 and IL-2, TNF or IFNg. The |

CD4+ events were defined as total memory or central memory according to expression of CCR7 and CD45RA. CD4+ cells with a TH1 phenotype were defined as memory cells that coexpressed CD69 and IL-2, TNF or IFNg. For PBMC only, CD4+ cells with a TH2 phenotype were defined as memory cells that co-expressed CD69 and IL-4 or IL-13, whereas TFH cells were defined as central memory CD4+ T cells that expressed CXCR5, ICOS and PD-1. TFH cells were further characterized as IL-21+, CD69+ or CD40L+, CD69+.

☒ Tick this box to confirm that a figure exemplifying the gating strategy is provided in the Supplementary Information.
